# Exploring Larval Axolotl Brain Development: Insights Into Developmental and Functional Constraints

**DOI:** 10.1111/ede.70034

**Published:** 2026-03-08

**Authors:** Laurent Houle, Olivier Larouche, Richard Cloutier

**Affiliations:** ^1^ Laboratoire de Paléontologie et Biologie évolutive Université du Québec à Rimouski Rimouski Quebec Canada; ^2^ Biology Department Western Carolina University Cullowhee North Carolina USA; ^3^ Center of Excellence on the Evolution of Life, Basin Studies and Applied Paleontology, Paleontological Research and Education Center Mahasarakham University Kantharawichai Maha Sarakham Thailand

**Keywords:** *Ambystoma mexicanum*, integration, modularity, neurobiology, ontogeny

## Abstract

Brain evolution in vertebrates has been conceptualized through two major hypotheses: the mosaic and concerted evolution models. The mosaic evolution model suggests that brain structures are primarily shaped by functional constraints, whereas the concerted evolution model emphasizes the role of developmental constraints. Our objectives in this study were (1) to describe brain shape and volume changes during Mexican axolotl (*Ambystoma mexicanum*) larvae development, and (2) to interpret possible functional and developmental constraints during post‐hatching brain maturation. A total of 77 larvae, spanning four developmental stages, were examined using 3D geometric morphometrics and volumetric measurements derived from iodine micro‐CT imaging. To understand the relationships among brain regions, we employed morphological integration and modularity analyses, providing a comprehensive assessment of changes in shape covariation patterns during post‐hatching development. Our results reveal that the telencephalon‐diencephalon boundary and the hypothalamus region exhibit a low level of morphological variation throughout larval development. This stability may influence the positioning of the coronal suture, a key feature in tetrapod skull morphogenesis. In contrast, sensory structures undergo significant changes. The olfactory bulbs and optic tectum display positive allometric growth during early post‐hatching development, transitioning to isometric growth at later stages. These shifts suggest an early developmental emphasis on sensory‐related brain areas, potentially driven by functional constraints. Results also revealed a general correspondence between brain region volume and total brain volume, which aligns with the concerted model. Modular, morphological integration, and volumetric analyses suggest that an interplay of functional and developmental constraints might be involved in axolotl brain development.

## Summary

- Six independent brain regions have been found in larval axolotls.
- Telencephalon–diencephalon boundary is stable in development.
- Evidence supports both concerted and mosaic brain evolution.

## Graphical abstract

We studied brain shape and volume variations during larval development of the Mexican axolotl. Modularity, morphological integration, and allometric regressions supported both mosaic and concerted evolution models, highlighting a complex interplay of constraints during axolotl ontogeny.

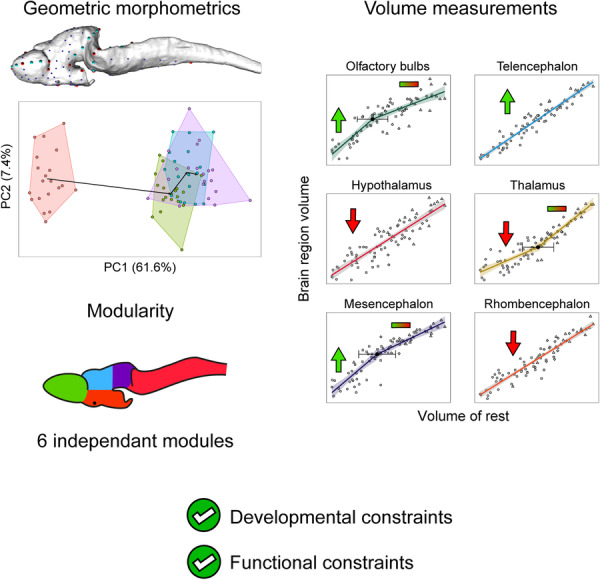

## 1 Introduction

Two main hypotheses have been proposed to explain the evolution of vertebrate brains: the mosaic and concerted evolution models. The mosaic model suggests that individual brain regions can evolve independently, with morphological differences driven by adaptive divergence in function and selective pressures (Barton and Harvey 2000). In contrast, the concerted model suggests that the brain evolves as an integrated unit, shaped primarily by global developmental constraints such as the timing and duration of neurogenesis (Finlay et al. 2001). To compare the relative support of these models, many studies have relied on how brain regions scale with total brain size, using allometric relationships between regional and whole‐brain volumes (e.g., Finlay and Darlington 1995; Finlay et al. 2001; Gonzalez‐Voyer et al. 2009; Gutiérrez‐Ibáñez et al. 2014; Montgomery et al. 2016). Under a concerted model, brain regions are expected to follow shared scaling trends with total brain size, reflecting strong developmental integration, whereas mosaic evolution is inferred when particular regions deviate from these trends or show patterns of co‐variation that are independent of overall brain size (Barton and Harvey 2000; Gutiérrez‐Ibáñez et al. 2014; Schumacher and Carlson 2022). These two hypotheses are not mutually exclusive, and their respective contributions to brain structure variation remain debated (Barton and Harvey 2000; D'aniello et al. 2019; Willemet 2020). While several studies support the mosaic evolution model across diverse vertebrate groups (Barton and Harvey 2000; Gómez‐Robles et al. 2014; Gonzalez‐Voyer et al. 2009), others find stronger evidence for concerted evolution (Dumitru and Frugård Opdal 2024; Finlay et al. 2001; Stearns 2018). In amphibians, both models were found to be involved in the evolution of brain size in anurans (Liao et al. 2015). While these two evolutionary models have been mostly contrasted at a macroevolutionary scale, intraspecific analyses using shape and volume variation should also be useful, given that part of the macroevolutionary patterns of covariation may arise from intraspecific variation.

The vertebrate brain is a complex organ that exhibits considerable disparity in shape and size and has been associated with the diversification of several vertebrate lineages (Balanoff et al. 2016; Barton et al. 1995; Pollen et al. 2007). Comparative analyses demonstrate that brain regions can be linked to organismal functions critical for survival (Gonzalez‐Voyer et al. 2016; Nieuwenhuys et al. 2014; Striedter 2005). For example, taxa highly reliant on visual and olfactory sensory information, such as sharks (Yopak and Lisney 2012) and insectivorous mammals (Barton et al. 1995), typically exhibit enlarged optic tecta and olfactory bulbs (Jerison 2012). Conversely, primates and marine mammals display a disproportionately enlarged telencephalon and cerebellum, associated with complex cognitive functions (Jerison 2012). Even intraspecifically, the relative size and shape of brain regions can also change during ontogeny in response to specific physiological and environmental demands. These changes can include drastic morphological changes, such as those occurring during metamorphosis or even seasonal variations (Kollros 1981; Lázaro et al. 2018; Yaskin 2011). Such great versatility of shape highlights the importance of studying the morphological evolution of the brain.

Several authors have observed a general trend of complexification of brain structure throughout vertebrate evolution (Hodos 1988; Hofman 1982; Jerison 2012). However, some groups like amphibians, coelacanths, and lungfishes exhibit relatively simple brain morphologies (Dutel et al. 2019; Northcutt 1986; Roth et al. 1993). Salamanders, in particular, retain a Bauplan similar to early tetrapods and thus can increase our understanding of the complex processes underlying vertebrate evolution (Bonett et al. 2022; Kawano et al. 2024; Schwarz et al. 2023). Among salamanders, the Mexican axolotl (*Ambystoma mexicanum*) is considered a valuable model species (Bölük et al. 2022; Vieira et al. 2020; Voss et al. 2009). Numerous studies have used axolotls as a model to address developmental and evolutionary questions (Denis et al. 2013; Hincapie Agudelo et al. 2021; Malacinski 1978), including many focused on brain structure and development (Amamoto et al. 2016; Feng et al. 2023; Fritzsch et al. 2005). Although some salamander groups, particularly plethodontids, have been associated with secondary brain simplification, this pattern is not observed in the axolotl (Roth et al. 1997), which retains functional and developmental features comparable to those of other vertebrates (Maddin et al. 2016; Roth and Walkowiak 2015).

Moreover, a key biological feature of the axolotl is its pedomorphosis, whereby individuals retain larval characteristics throughout life by avoiding metamorphosis (Gould 1977; Shaffer 1993). This condition is tightly linked to extremely low circulating levels of thyroid hormones (TH; Brown and Cai 2007), which are major regulators of vertebrate brain development, neurogenesis, and neural differentiation (Tata 2006). Because TH plays a central role in orchestrating early brain maturation in most vertebrates (Mourouzis et al. 2020), the persistent TH deficiency in axolotls provides a unique context in which to examine brain development in the relative absence of typical endocrine triggers.

This study has two main objectives: (1) to describe patterns of post‐hatching morphological development of the brain of *A. mexicanum* larvae, and (2) to examine whether the variation of the shapes and relative volumes of brain regions could suggest effects of functional and developmental constraints on developing brain regions. To address these objectives, we combine geometric morphometrics analyses with volumetric measurements (Slice 2007; Zelditch et al. 2012). Geometric morphometrics provides a powerful statistical framework to capture complex shape variation, quantify relative size differences, and assess morphological integration and modularity (Zelditch et al. 2012). Morphological integration refers to the strength of correlations among morphological traits, whereas modularity describes the tendency of an organism to be subdivided into partially independent groups of traits called modules (Olson and Miller 1999). Quantifying morphological integration and modularity patterns promotes a better understanding of morphological evolution and development since it allows a deep investigation of the covariation among sets of morphological regions. These two concepts have also been linked to contrasting views on the relative influence of the concerted and mosaic models: high modularity is generally interpreted as support for the mosaic model, reflecting a predominant role of functional constraints (Gómez‐Robles et al. 2014), whereas strong morphological integration is more consistent with the concerted model and implies a greater influence of developmental constraints (Gómez‐Robles et al. 2014; Mitteroecker and Bookstein 2008). More specifically, we examine two main hypotheses. Our first hypothesis is that brain morphology undergoes its greatest changes immediately after hatching, before stabilizing during later development. For the second hypothesis, we expect that both functional and developmental constraints contribute to the development of the axolotl brain, which would be consistent with patterns observed in other vertebrates (Moore and DeVoogd 2017; Noreikiene et al. 2015).

## 2 Materials and Methods

### 2.1 Sample and Husbandry

We analyzed 80 post‐hatching larvae of *A. mexicanum* collected at four developmental stages (47, 50, 52, 54; Nye et al. 2003). All larvae originated from a single clutch (one male and one female leucistic adults) produced at Carleton University (Ottawa, Canada) under Canadian Council on Animal Care protocol #102951. Adults were obtained from the *Ambystoma* Genetic Stock Center (University of Kentucky, USA). One adult specimen was included as representative of the adult brain shape and volume conditions (University of Calgary Animal Care Committee protocol AC15‐0020).

Eggs were incubated at 18°C in a 40% Holtfreter's solution. After hatching, larvae were reared in 20% Holtfreter's at 19°C under a 12:12 light:dark cycle and fed daily (earlier stages were fed baby brine shrimp, while later stages were fed bloodworms). Individuals were isolated during forelimb bud formation to prevent injury owing to limb bud biting behavior and to ensure accurate staging. Once in their individual compartments, the specimens were fed a diet of bloodworms once per day. Specimens were staged daily using the Bordzilovskaya et al. (1989) and Nye et al. (2003) normal tables of development. Four developmental stages were selected: 47, 50, 52, and 54 according to Nye et al. (2003). These stages were selected to cover most of the first month of the free‐foraging larval period and the beginning of the ossification of the skull roof. A sample of 20 specimens was euthanized on the same day for each stage using a 4% tricaine methanesulfonate (MS‐222) solution (Sigma‐Aldrich E10521, St. Louis, MO) buffered using sodium bicarbonate (pH of 7.1).

Specimens were fixed in a 4% neutral‐buffered formalin (Thermo Fisher Scientific SF1004, Waltham, MA) solution for 4 h. After fixation, larvae were washed in distilled water and dehydrated gradually using 15%, 30%, and 50% (2 h in each solution) ethanol solutions, and then stored in 70% ethanol. Among the specimens that were euthanized, three (one from stage 50 and two from stage 52) were removed from downstream analyses because their brains appeared to be damaged due to handling (Table 1). The adult specimen was euthanized in 5% MS‐222; the specimen was kept in the solution until cardiac activity ceased entirely (approx. 1 h), and was then fixed in 10% neutral‐buffered formalin for 24 h.

**Table 1:** Description of the developmental stages and corresponding days post‐hatching for *Ambystoma mexicanum* larvae included in the sample.

Developmental stage (Nye et al. 2003)	Day post‐hatching	Sample size for analyses
47	10	20
50	16	19
52	22	18
54	28	20
Adult	—	1

### 2.2 Staining and Imaging Procedures

All larvae were stained in a solution composed of 0.3% phosphotungstic acid and 70% ethanol following Metscher (2009). The adult specimen was stained in an 11.75% iodine solution (I_2_KI) for 5 days. Specimens were scanned using a Bruker Skyscan 1173 at a resolution of 8.1 μm. For all the scans of larvae, the exposure time was usually 1325 ms, the voltage and current were set at 77 kV and 79 µA, respectively. The reconstruction of images was done using the NRecon software (Bruker Corporation).

The segmentation of the brains was performed using the Dragonfly software v.2022.2 (Dragonfly 2022). A standardization of the grayscale domain of each image was performed to eliminate high greyscale domain variation across images of each specimen (gradient domain fusion filter in Dragonfly). A deep learning model was created to help the segmentation process. The model had a U‐Net architecture using a 2.5D method (adjusting segmentation using the two previous and next images), five layers, and 112 pixels starting squares. The model was trained using between 10 and 50 images per specimen from various areas of the brain. The first draft of the segmentation process was produced by the model. Each image was then manually adjusted by the same observer (L. H.). The region of interest from the segmentation process was converted into a 3D surface mesh and then smoothed to reduce imperfections (Laplacian smoothing).

### 2.3 Geometric Morphometrics

**Figure 1:**
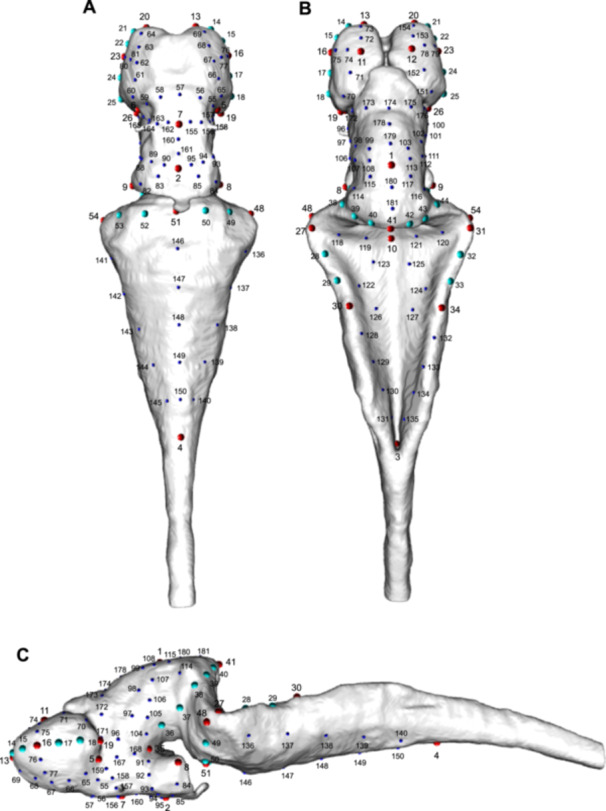
Ventral (A), dorsal (B), and lateral (C) views of the landmarks placed on the brain of *Ambystoma mexicanum* larvae. Large red spheres represent fixed landmarks, large cyan spheres represent curve semilandmarks, and small dark blue spheres represent surface semilandmarks. [Color figure can be viewed at wileyonlinelibrary.com]

A sample of 28 fixed landmarks, 26 curve semilandmarks, and 127 surface semilandmarks was used to capture the shape information of the brain (Figure 1, Supporting Information S1: Table 1). The landmarks were placed using the Checkpoint software (Stratovan Corporation). All shape analyses were performed in R (v.4.4.3) (R Core Team 2024). An automated procedure was used to apply surface semilandmarks on meshes using the placePatch function in the “Morpho” package v.2.12 (Schlager 2017). We used the findMeanSpec function from the “geomorph” package v.4.0.8 (Baken et al. 2021) to identify the specimen closest to the mean shape, which was then selected as the atlas for automatic surface semilandmark placement. A generalized Procrustes analysis was performed on the landmark coordinates in order to control for differences in scaling, orientation, and positioning of the configurations of landmarks (Rohlf and Slice 1990).

A fluctuating asymmetry analysis was performed to quantify and exclude the shape changes resulting from bilateral asymmetry in the data (Klingenberg et al. 2002). To assess the repeatability of morphometrics data collection, measurement error was calculated following the method developed by Collyer and Adams (2024) using the measurement.error function from the “RRPP” package v.2.0.4 (Collyer and Adams 2019). The total measurement error represented 5.6% of the entire shape variation, and 94.3% of this amount represented random measurement error. Systematic measurement error was not significant (*R* ^2^ = 0.0007, *Z* = −0.271, *p* = 0.612) and was not significantly different among developmental stages (*R* ^2^ = 0.003, *Z* = 1.54, *p* = 0.072). The variation associated with measurement error was removed from the shape data using the fluctuating asymmetry analysis.

A Procrustes ANCOVA was used to examine the influence of (log) centroid size and developmental stages on the symmetrical component of shape variation, as well as to examine the interaction between the two independent variables. A Procrustes linear regression was performed to examine whether shape variation is influenced by size variation (Collyer et al. 2015). Three‐dimensional deformation grids comparing stages were used to interpret size‐related shape changes. Since the effect of size on shape was significant and substantial (*F*_1,77_ = 56.738, *Z* = 3.472, *R* ^2^ = 0.612, *p* = 0.001), two datasets were generated, one that preserves the effect of size on shape and one that controls for allometry. For the latter shape dataset, the residuals from the Procrustes linear regression between shape and size were added to the consensus shape.

Principal component analyses (PCA) were performed to compare the morphospace occupation of developmental stages while including or excluding the effect of size. Three‐dimensional deformation grids were used to investigate shape changes along the principal components. Procrustes distance histograms were performed to evaluate if the variation among stages was associated with higher or lower Procrustes distances than those within each stage. Procrustes ANOVAs and pairwise comparisons were used to investigate shape differences among developmental stages for both datasets (i.e., with and without the effect of size on shape). Pairwise comparisons were made for the Procrustes distances among mean shape and for the variance among stages using the pairwise function from the “RRPP” package v.2.0.4 (Collyer and Adams 2019).

### 2.4 Volumetric Measurements

The brain of each specimen was numerically dissected into six morphological partitions (olfactory bulbs, telencephalon, hypothalamus, thalamus, mesencephalon, and rhombencephalon) using the Dragonfly software. The limits of each partition followed Lazcano et al. (2021; see Supporting Information S1: Figure 1). Specifically, the end of the telencephalon lobes was used as an anatomical landmark to separate the telencephalon and hypothalamus. The anterior end of the lateral ventricle was used as the delineation between the olfactory bulb and the rest of the telencephalon. The diencephalic‐mesencephalic boundary was taken to be where the hypothalamus separates from the rest of the brain (slightly anterior to the delineation between the hypothalamus ventralis and dorsalis). The posterior end of the rhombencephalon was considered to be at the closure of the medulla oblongata. The volume of each partition was measured using the labeled voxels of the region of interest in Dragonfly.

The statistical methods applied to the volumetric data consisted of three main analyses: (1) allometric regressions of brain region volumes, (2) mean log_10_‐shape ratio (LSR) comparisons, and (3) a volumetric data PCA. A model selection approach was used to produce a regression model between the volume of each brain region and the total brain volume minus the volume of that specific region. Both ordinary least squares (OLS) and piecewise regressions were computed for each brain region. Piecewise regressions consist of a model composed of several (two or more) linear regressions associated with different segments of data (Muggeo 2003). The best model was selected based on the results of an extra sum of squares F‐test between the OLS and the piecewise model for each brain region. Akaike Information Criterion (AIC) values were also considered in the model selection. Assumptions for the linear regression were assessed visually. The piecewise models were produced using the “segmented” package v.2.1‐3 (Fasola et al. 2018). This approach allowed for an investigation of the possible negative or positive allometric growth of individual brain regions, and to examine possible allometric shifts during development (Kilmer and Rodríguez 2017). A LSR standardization was also applied to the volume data (Mosimann 1970) to compare the relative volume of brain regions among developmental stages. This approach involves calculating a ratio between the volume of each individual region and the geometric mean across all volumes, and then applying a logarithmic transformation to this ratio (Mosimann 1970). A permutation one‐way ANOVA and permutation pairwise *t*‐tests were performed for each volume partition to test if there was a difference in the volume LSR among developmental stages. A Bonferroni correction was applied to the *p* values of the permutation pairwise *t*‐tests. A PCA was performed on the LSR data to verify if shape and volume data were correlated. To test the fit between brain shape and volume PCA scores, a Mantel test was used between each of the shape datasets (including and excluding the effect of size on shape) scores and the volume PCA scores using the “vegan” package v.2.6‐6.1 (Oksanen 2016).

### 2.5 Morphological Integration and Modularity Analyses

The datasets with and without allometric effects on shape were both analyzed for patterns of morphological integration and modularity. Individuals from different developmental stages were analyzed separately. We compared six different a priori modularity hypotheses (Table 2, Supporting Information S3). The signal of modularity was estimated using the covariance ratio (CR) method (Adams 2016) and the maximum‐likelihood approach (EMMLi v.0.0.3) developed by Goswami and Finarelli (2016). The CR coefficient represents the ratio between the covariances among modules and the within‐module covariances based on covariance matrices over all specimens and associated with shape variables of each module (Adams 2016). To assess the strength of the modular signal, the observed value is compared to a distribution of random CR values (null hypothesis) (Adams 2016). An effect size was also estimated, where increasingly negative values correspond to a stronger modular signal (Adams and Collyer 2019). The effect size can also be used in pairwise comparisons to determine if the strength of modularity differs statistically among hypotheses (Adams and Collyer 2019). This method was performed using the modularity.test and compare.CR functions from the “geomorph” package v.4.0.8 (Baken et al. 2021). The EMMLi analysis, a maximum likelihood‐based method, compares correlation coefficients between and within modules and estimates a model log_10_ likelihood for each hypothesis (Goswami and Finarelli 2016). To select the most supported hypothesis, EMMLi uses the corrected AIC (AICc) (Goswami and Finarelli 2016). Using this method, the hypothesis with the lowest AIC score would represent the best‐supported model (Goswami and Finarelli 2016). This method also tests if the within‐module correlations are equal or unequal across all modules considered in a hypothesis, as well as if the between‐module correlations are equal or unequal (Goswami and Finarelli 2016). Consequently, four models are compared for each hypothesis of three or more modules, while two models are compared for a two‐module hypothesis (Goswami and Finarelli 2016).

**Table 2:** Name and description of hypotheses used in modularity analyses on *Ambystoma mexicanum* larvae.

Hypothesis	Description	Number of modules	
Hypothesis 1	[Dorsal landmarks] and [ventral landmarks]	2	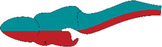
Hypothesis 2	[Forebrain + hindbrain] and [midbrain]	2	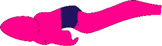
Hypothesis 3	[Forebrain], [midbrain], and [hindbrain]	3	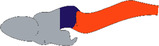
Hypothesis 4	[Telencephalon], [diencephalon], [mesencephalon], and [rhombencephalon]	4	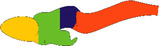
Hypothesis 5	[Olfactory bulbs], [telencephalon], [diencephalon], [mesencephalon], and [rhombencephalon]	5	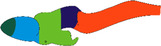
Hypothesis 6	[Olfactory bulbs], [telencephalon], [hypothalamus], [thalamus], [mesencephalon], and [rhombencephalon]	6	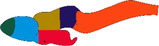

All morphological integration analyses were implemented using the “geomorph” package v.4.0.8 (Baken et al. 2021). To assess the level of morphological integration for each modularity hypothesis, partial least‐squares analyses were performed (integration.test function) (Collyer et al. 2015). Using this method, a low partial least squares (PLS) value represents a low level of inter‐module integration (Collyer et al. 2015). In addition, regressions between the variance of partial warps and their associated bending energy (globalIntegration function) were produced to assess the strength of integration of the shape of each developmental stage (Bookstein 2015). The relative eigenvalue index (Vrel) was also used to compare the level of integration among developmental stages using their associated effect size (Conaway and Adams 2022).

## 3 Results

### 3.1 Description of Shape and Volume Changes in Post‐Hatching Axolotls

**Figure 2:**
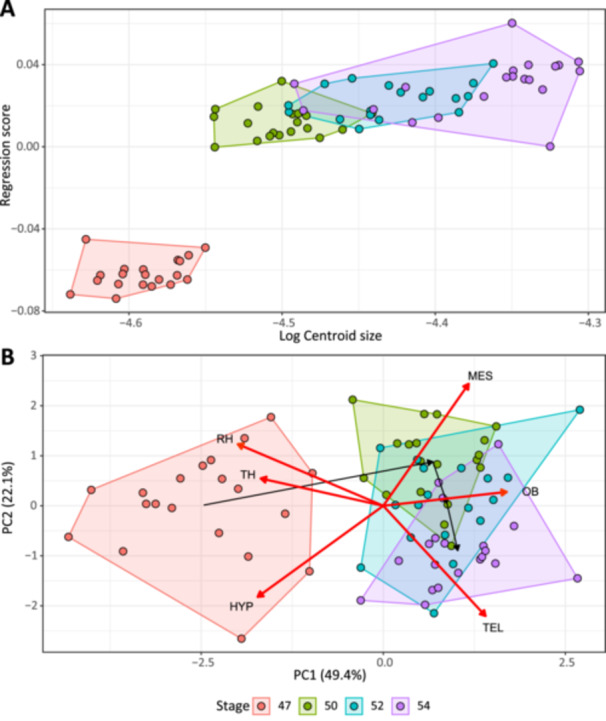
Regression analysis of 3D brain shape (regression scores) against log_10_‐centroid size (A) and volumetric data PCA (B) showing differences among four developmental stages (polygons) of *Ambystoma mexicanum* larvae. Red arrows in plot C represent loadings of volumetric data. Percentages of explained variation are provided in parentheses for each PCA axis. HYP, hypothalamus; MES, mesencephalon; OB, olfactory bulbs; RH, rhombencephalon; TEL, telencephalon; TH, thalamus. [Color figure can be viewed at wileyonlinelibrary.com]

The centroid size of brain coordinates had a significant effect on shape and accounted for 61.2% of the total variation (*F*_1,75_ = 38.439, *Z* = 5.469, *R* ^2^ = 0.612, *p* = 0.001). This substantial effect can mostly be attributed to the divergence between stage 47 and the three other stages (Figure 2). The Procrustes ANCOVA showed that there were no significant differences among slopes for the four developmental stages concerning the relationship between brain shape and centroid size (*F*_3,69_ = 1.258, *Z* = 1.302, *R* ^2^ = 0.019, *p* = 0.092).

**Figure 3:**
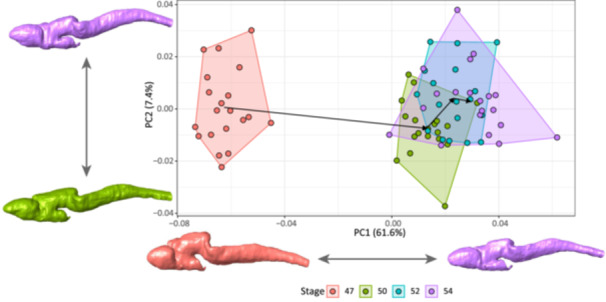
Size‐included shape data PCA showing differences among four developmental stages (polygons) of *Ambystoma mexicanum* larvae. Brain meshes illustrate the shapes at the positive and negative extremes of each principal component axis. [Color figure can be viewed at wileyonlinelibrary.com]

**Figure 4:**
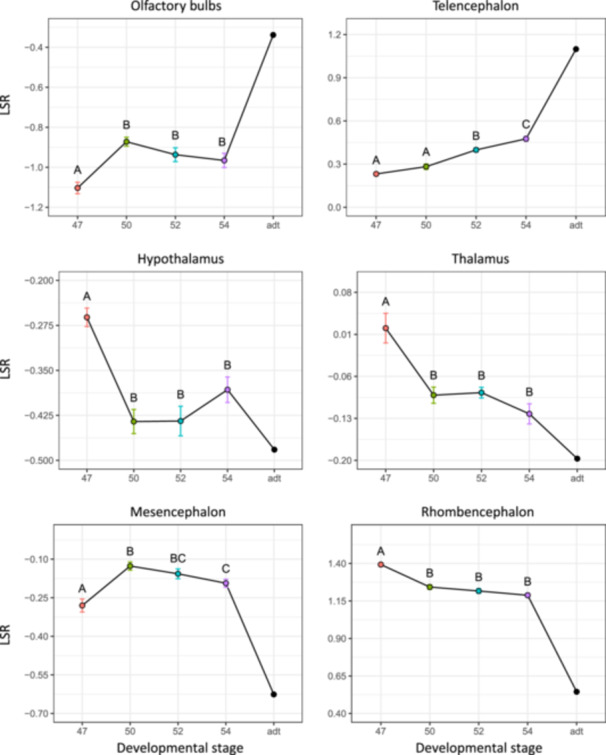
Comparison of the mean log_10_‐shape ratio (LSR) of six volume partitions of the brain of *Ambystoma mexicanum* larvae across four developmental stages (stages 47, 50, 52, and 54) and one adult specimen (adt). Error bars represent the standard error, and letters refer to permutation pairwise *t*‐tests results. [Color figure can be viewed at wileyonlinelibrary.com]

**Figure 5:**
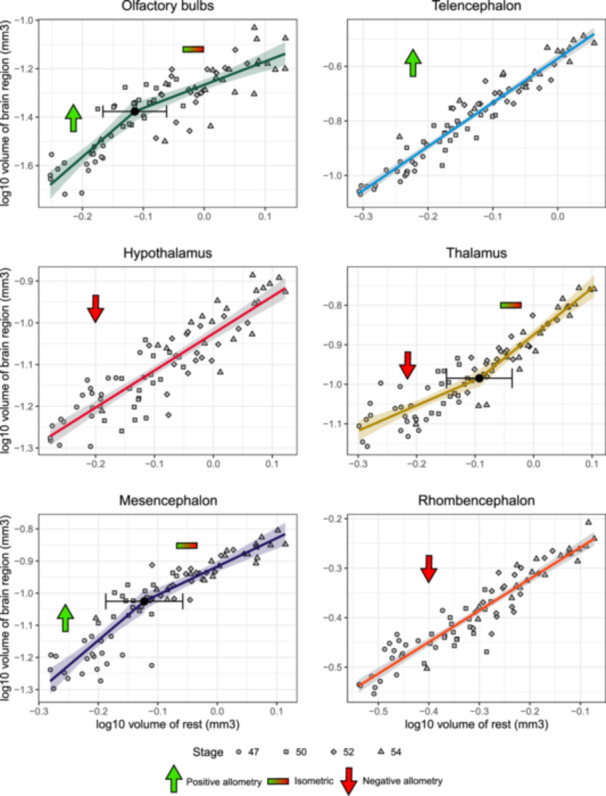
Best supported regression model comparing the volume of each brain region with the total volume of the brain minus the volume of the region (log_10_ transformation applied to x and y) for *Ambystoma mexicanum* larvae. Shaded areas represent the 95% confidence interval of each regression line. Black dots indicate a breakpoint in the regression model, and the error bar represents its 95% confidence interval. [Color figure can be viewed at wileyonlinelibrary.com]

For the dataset including allometry, the PCA and Procrustes ANOVA showed a visible difference between stage 47 and the other stages, and also differences, albeit of a smaller magnitude, among stages 50, 52, and 54 (Figure 3 and Supporting Information S1: Table 2). The Procrustes variance among developmental stages did not significantly differ (Supporting Information S1: Table 2). The Procrustes distance histogram shows that the within‐stage variation is composed mostly of small distances, while the among‐stage variation consists of a wide range from small to large distances (Supporting Information S1: Figure 2). This diversity of small and large Procrustes distances might reflect the substantial divergence between stages 47 and 50, and the much smaller differences observed among the other stages (Figure 3). These differences account for most of the explained variation of the first principal component of the shape PCA (Figure 3). The shape deformation associated with the transition from stage 47 to 50 is similar to the size‐related shape changes observed from the shape allometric regression (Supporting Information S1: Figures 3 and 4). These changes consist of an elongation of the olfactory bulbs, a ventral depression of the mesencephalon, and an anterior and lateral constriction of the medulla oblongata (Figure 3, Supporting Information S1: Figure 4). This last region also shows a dorsal elevation of its posterior end. From stages 50 to 52, shape changes are similar to those from stages 47 to 50, but with a much smaller magnitude (Supporting Information S1: Figure 4). The transition between stages 52 and 54 is mostly associated with the posterior retraction of the ventral section of the medulla oblongata (Supporting Information S1: Figure 4). Using the dataset where the effect of size on shape is removed, the patterns observed in the deformation grids are similar, but with lower magnitudes (Supporting Information S1: Figure 5). Developmental stages still explain 25.2% of the total variation, and there is still no significant difference in Procrustes variance across stages (Supporting Information S1: Table 3).

The Mantel tests showed that volume PCA was correlated with both the dataset including allometry (*p* < 0.001, *r* = 0.665) and the one excluding allometry (*p* < 0.001, *r* = 0.265). Both the volume PCA and mean LSR values indicated that stage 47 was substantially different than other developmental stages (Figures 2 and 4). Volume LSR of stage 47 for all partitions differed significantly from the three other stages (Figure 4). Stage 47 was associated with a small relative volume for the olfactory bulbs, telencephalon, and mesencephalon, and large volumes for the hypothalamus, thalamus, and rhombencephalon (Figure 4). From stages 47 to 50, the volume of brain regions exhibited drastic changes: the olfactory bulbs and mesencephalon increased in relative volume while the volumes of the hypothalamus, thalamus, and rhombencephalon decreased (Figure 4). From stages 50 to 54, the volume of the olfactory bulbs, the thalamus, hypothalamus, and rhombencephalon did not differ significantly (Figure 4). During that same period, the telencephalon progressively increases in relative size (Figure 4) while the volume of the mesencephalon finally decreases at stage 54 (Figure 4). A decrease in the relative volume of the thalamus, hypothalamus, mesencephalon, and rhombencephalon, along with an increase in the relative volume of the olfactory bulbs and telencephalon, is observed when comparing stage 54 to adult data.

### 3.2 Investigating Support for a Mosaic or Concerted Evolution of Brain Regions

Allometric regressions of volumetric measurements (Supporting Information S1: Table 4) indicated that the olfactory bulbs, thalamus, and mesencephalon are associated with allometric shifts with a breakpoint approximately at stage 50 (Figure 5 and Supporting Information S1: Table 5). Stage 47 is associated specifically with a positive allometric growth for the olfactory bulbs and the mesencephalon and a negative allometric growth for the thalamus. The telencephalon exhibits positive allometry, while the hypothalamus and rhombencephalon grew at a lower rate than the rest of the brain (Figure 5 and Supporting Information S1: Table 5). While differences among brain regions are present, the predictive capability of linear models was generally strong (*R* ^2^ value ranging from 0.777 to 0.922; Supporting Information S1: Table 5).

**Figure 6:**
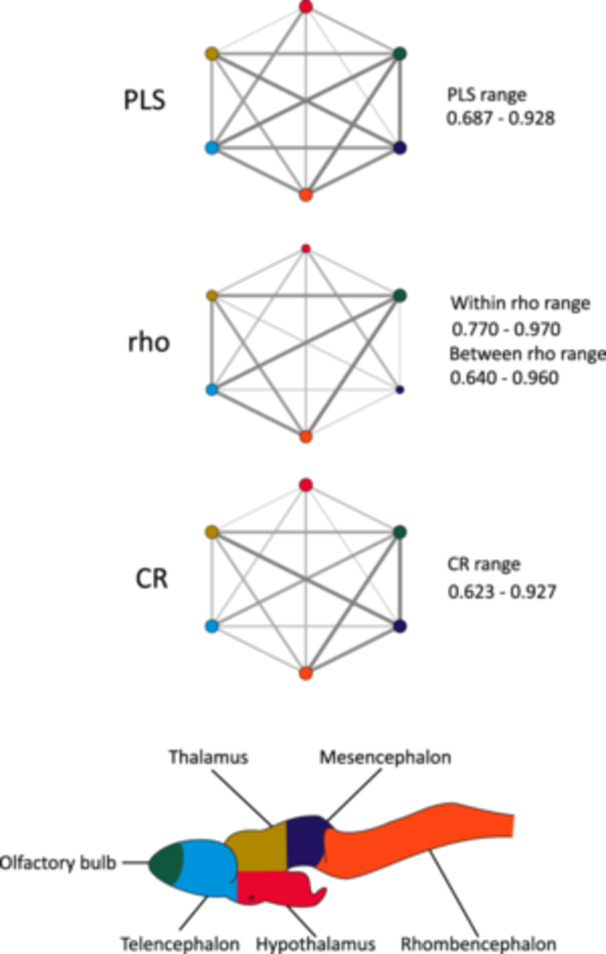
Network representations of the strength of morphological integration from the covariance ratio (CR), the EMMLi (rho), and the partial least squares (PLS) analyses for the brain shape of *Ambystoma mexicanum* larvae. Edge width in each network represents the strength of the association between two modules. Note that the edge width is based on values within each analysis (i.e., not directly comparable between networks). For the EMMLi analysis, the edge width represents the correlation between two modules, and the size of nodes represents the relative within‐module correlation. [Color figure can be viewed at wileyonlinelibrary.com]

The results of modular analyses for the datasets, including and excluding allometry, yielded the same general conclusions qualitatively. Therefore, only the results associated with the shape dataset controlling for allometry are presented in the main text (see Supporting Information S2 for the allometry included results). The best‐supported hypotheses using the CR method on all larvae were Hypotheses 4–6, which all showed similarly strong modular signals with effect sizes ranging from −8.5 to −8.9, whereas Hypotheses 1–3 exhibited much weaker values, between −3 and −5 (Table 3). The differences among these three hypotheses were associated with the separation of the olfactory bulb from the rest of the telencephalon between Hypotheses 4 and 5, and the separation of the thalamus and hypothalamus for Hypotheses 5 and 6. Specific investigation of CR values across modules of these three hypotheses revealed that the hypothalamus is systematically less integrated with other modules (Figure 6 and Supporting Information S1: Figure 6). This may be linked to the lack of shape variation associated with this region. The olfactory bulb, when considered as a separate module, seems to be generally more integrated than the rest of the telencephalon with other modules (Supporting Information S1: Figure 6).

**Table 3:** Results of morphological integration analyses using partial least squares (PLS) and modularity analyses using the covariance ratio (CR) for the six a priori hypotheses for the brain of *Ambystoma mexicanum* larvae.

	CR method			Morphological integration		
Hypothesis	CR	*p* value	*Z*	PLS	*p* value	*Z*
1	0.901	0.002	−3.934	0.923	0.001	4.304
2	0.886	0.001	−5.341	0.900	0.001	5.276
3	0.878	0.001	−5.769	0.917	0.001	8.519
4	0.816	0.001	−8.540	0.885	0.001	9.509
5	0.796	0.001	−8.872	0.887	0.001	7.096
6	0.776	0.001	−8.736	0.847	0.001	7.041

*Note:* Hypotheses are described in Table 2.

Using the EMMLi analysis, Hypothesis 6 was the best‐supported hypothesis (with variations observed in the strength of correlations both within and between modules), exhibiting the lowest AIC value across all tested models (Table 4). This best‐fitting model indicates that correlation strengths are not uniform across all modules (within correlations) or between module pairs (between correlations). The evaluation of these correlations for Hypothesis 6 revealed a somewhat different pattern compared to the CR method. The hypothalamus is less correlated with other modules, but the mesencephalon also exhibits this pattern (Figure 6). Furthermore, in this analysis, the olfactory bulbs showed strong correlations with the telencephalon, thalamus, and rhombencephalon, whereas correlations between the olfactory bulbs and the hypothalamus remained weak. The hypothalamus stands out as being the least integrated region using both the EMMLi and CR methods.

**Table 4:** Results of modularity hypotheses comparison using the EMMLi methods for the six a priori hypotheses for the brain of *Ambystoma mexicanum* larvae.

Hypothesis	*K*	AICc	ΔAICc
1	4	833,281.941	130,544.140
2	4	885,704.137	182,966.335
3	7	809,423.868	106,686.066
4	11	732,786.505	30,048.703
5	16	724,795.690	22,057.889
6	22	702,737.802	0.000

*Note:* Hypotheses are described in Table 2.

Taken individually, each developmental stage exhibited a significant modular signal using the CR approach (Supporting Information S1: Table 6). While Hypothesis 1 had an effect size that differed significantly from that of hypotheses with more complex modular organization (such as Hypotheses 4–6), nonsignificant results were found among all other hypotheses (Supporting Information S1: Table 7). This may be related to a lack of statistical power owing to insufficient sample sizes within each developmental stage. Hypothesis 6 was best supported by the EMMLi analyses for every developmental stage (Supporting Information S1: Table 6). These results seem to indicate that the modular signal was strong and that the modularity pattern is generally conserved among developmental stages.

Concerning morphological integration, the brain shape of *A. mexicanum* larvae can be considered as disintegrated (slopes between −1 and 0) following Bookstein's (2015) concept (Supporting Information S1: Table 8). In this context, disintegration can be interpreted as a major contribution from small‐scale variation on the brain shape, whereas integration would be associated with a major contribution from large‐scale variation. Stage 47 is the least integrated developmental stage (Supporting Information S1: Table 8). The pairwise PLS analyses indicate an intermediate level of integration among brain regions (Table 3). The level of morphological integration estimated using the relative eigenvalue index does not vary significantly among developmental stages (Supporting Information S1: Table 9). Pairwise comparisons of PLS effect sizes among hypotheses revealed that Hypotheses 3–6 had a significantly lower level of inter‐module integration than Hypotheses 1 and 2 (Supporting Information S1: Table 10). These results, in association with the similar Procrustes variance comparison, indicate that the level of morphological integration appears to be fairly constant among developmental stages.

## 4 Discussion

This study combines analyses of shape and volumetric data to characterize the brain development of axolotl larvae across four developmental stages. Our results show that the shape and relative volumes of brain regions change during development, providing insights into the relationship between these observed brain patterns and skull development. Additionally, our analyses of modularity and morphological integration, combined with volumetric analyses, suggest that early brain development of post‐hatching axolotl is shaped by both functional and developmental influences.

### 4.1 Evidence for the Role of Developmental Constraints

The vertebrate skull roof consists of bones originating from different embryonic tissues, and although the contributing tissues vary among lineages, their proper arrangement depends on signaling centers that may be conserved across vertebrates (Maddin et al. 2016; Teng et al. 2019). In axolotls, the pattern of cranial neural crest (CNC) contributions to the bony skull is similar to that observed in amniotes (Piekarski et al. 2014). Specifically, the neurocranium of amniotes and axolotls shows a distinct separation at the coronal suture, with CNC‐derived bony structures located anteriorly, and non‐CNC‐derived bony structures positioned posteriorly (Maddin et al. 2016; Piekarski et al. 2014). In amniotes, the boundary between the telencephalon and diencephalon (near the optic chiasm) has been suggested as a key determinant of the frontoparietal suture location (Teng et al. 2019). Our findings on the relative shape and volume stability of the hypothalamus region are consistent with observations in squamates after mid‐stage development (Ollonen et al. 2024). These results may support the hypothesis that this region plays a role in determining the location of the frontoparietal suture in tetrapods (Atkins et al. 2020), as these two paired bones begin to ossify between stages 47 and 50 in Nye et al.'s (2003) table. This interpretation is consistent with the growing evidence that embryonic brain signaling centers play a crucial role in guiding suture formation (Teng et al. 2019). However, further work on suture specification cues is needed to better understand the dynamics of the coronal suture formation and the role of the telencephalon‐diencephalon boundary.

### 4.2 Evidence for the Role of Functional Constraints

The olfactory and visual systems have been revealed as highly important in feeding success in amphibian larvae (Rot‐Nikcevic et al. 2005). The larval period is also a moment of rearrangement and maturation of several visual, olfactory, and vomeronasal structures (Cuny and Malacinski 1986; Eisthen 2000; Eisthen et al. 1994). Early hatched predatory larvae, such as the axolotl, use several sensory mechanisms for foraging and sensing predators (Różański and Żuwała 2020). The axolotl eyes are well developed at hatching (Suetsugu‐Maki et al. 2012; Tesařová et al. 2022), but the lens and the retina are associated with several modifications until the late juvenile phase (Cuny and Malacinski 1986). Rearrangement of olfactory structures are also observed in salamander larvae (Eisthen 2000; Eisthen et al. 1994; Różański and Żuwała 2020). Such reorganization might also be associated with brain morphological variation. For instance, visual experience in fishes has been associated with the relative size of the optic tectum (Hall and Tropepe 2018; Soares et al. 2005). In our results, olfactory bulbs and the mesencephalon (including the optic tectum) seem to exhibit a similar relative volumetric growth in post‐hatching axolotls with a positive allometry in stage 47, followed by an isometric growth in later stages. Notably, among all brain regions, only the mesencephalon showed positive allometric growth during the developmental period examined, while also being characterized by a smaller relative volume in adults. This might be the result of a stronger investment in sensory system‐related structures in the early free‐swimming larval life. Therefore, the differential growth of the optic tectum and olfactory bulbs may be influenced by visual and olfactory input acting as a functional constraint. Furthermore, the specific grouping of regions recovered in the best‐supported modularity hypothesis highlights that certain sensory structures tend to covary more closely with one another, consistent with their shared roles in early larval behavior and sensory processing. The strong correlations identified by the EMMLi analysis between the olfactory bulbs and the telencephalon, thalamus, and rhombencephalon may reflect early integration among sensory‐processing regions involved in odor detection, sensory evaluation, and foraging‐related behaviors (Butler and Hodos 2005). In contrast, the weak correlation between the olfactory bulbs and the hypothalamus could suggest that interoceptive or valence‐related pathways may not yet be strongly integrated at these early developmental stages, or that these connections mature later in ontogeny. Taken together, these developmental changes likely explain the morphological differences observed among larval stages, with the marked transition between stages 47 and 50 reflecting a key phase of sensory and neural reorganization.

### 4.3 Morphological Integration and Modularity Patterns Support Both the Concerted and Mosaic Models

Morphological integration is generally viewed as supporting the concerted model of brain evolution (Gómez‐Robles et al. 2014; Mitteroecker and Bookstein 2008), whereas high modularity is typically interpreted as evidence for the mosaic model (Gómez‐Robles et al. 2014). Both the mosaic and concerted models have been proposed to explain brain evolution in vertebrates (Gutiérrez‐Ibáñez et al. 2014; Hoops et al. 2017; Moore and DeVoogd 2017). For instance, Watanabe et al. (2021) found both integrated and modular patterns shaping the evolution of the brain of avian and non‐avian coelurosaurians. Evidence supporting the mosaic model has been observed in specific contexts and vertebrate groups (Barton and Harvey 2000; Gómez‐Robles et al. 2014; Hager et al. 2012). For example, in teleosts, observed mosaic changes of specific brain regions associated with electrosensory systems were considered as support for independent size changes related to functional constraints (Schumacher and Carlson 2022). The modular organization of the brain has been suggested to promote mosaic evolution in chimpanzees and humans (Gómez‐Robles et al. 2014). Studies of salamander skulls have revealed high levels of modularity (Bon et al. 2020; Fabre et al. 2020), which is consistent with some of our observations in the axolotl brain. Our results indicate that visual and olfactory demands are associated with patterns of brain shape and volume, and the high level of modularity observed further suggests that mosaic processes may have contributed to axolotl brain evolution.

Our results also provide evidence consistent with the concerted model. Although volumetric analyses revealed distinct regional trends, brain region volumes still generally scale with overall brain size. Additionally, while the brain shape does show limited support for global integration, the brain still exhibits a fair amount of integration among modules (Figure 6). These results suggest that developmental constraints may be affecting all brain regions, lending some support for the concerted model.

In conclusion, our study provides new insights into the brain development of axolotl larvae, highlighting significant changes in brain shape and volume between stages 47 and 50. Our results suggest that both developmental and functional constraints might play a role in the developing brain of axolotls. Additionally, the stability of the telencephalon‐diencephalon boundary and hypothalamus volume throughout development may influence the positioning of the frontoparietal suture, as proposed in other tetrapods. These findings underscore the interplay of evolutionary and developmental factors in shaping brain and skull development in vertebrates. However, future studies might expand the developmental sequence of brain change to allow a more comprehensive understanding of the morphological variation in the developing brain of salamanders.

## Conflicts of Interest

The authors declare no conflicts of interest.

## Supporting information

Supplemental results 1.

Supplemental results 2.

Supplemental results 3.

## Data Availability

Raw landmark and volumetric data and the morphosource ID of each specimen are available from a GitHub repository (https://github.com/LaurentHoule03/Raw_data_Axolotl_Brains.git). Reconstructed images are available on the Morphosource website.
