# Supplementary material for: Exploring Larval Axolotl Brain Development: Insights Into Developmental and Functional Constraints
Source: Evol Dev. 2026 Mar 8;28(1):e70034. doi: 10.1111/ede.70034 (PMC12968592; doi:10.1111/ede.70034)
Supplement: Supplementary file 1 — Supplemental results 1. [file EDE-28-e70034-s003.docx]

**Title**

Exploring Larval Axolotl Brain Development: Insights into Developmental and Functional Constraints

**Authors**

Laurent Houle^1*^, Olivier Larouche^2^ and Richard Cloutier^1,3^

**Affiliations**

^1^ Laboratoire de Paléontologie et Biologie évolutive, Université du Québec à Rimouski, 300 Allée des Ursulines, Rimouski, Quebec G5L 3A1

^2^ Biology Department, Western Carolina University, Cullowhee, NC, 28723

^3^ Center of Excellence on the Evolution of Life, Basin Studies and Applied Paleontology; Paleontological Research and Education Center, Mahasarakham University, Maha Sarakham 44150, Thailand

^*^Corresponding author: laurent_houle@uqar.ca

Supporting information

Table 1. Description of landmarks used on the brain of *A. mexicanum* larvae. curveSM = curve semilandmark. surfaceSM = surface semilandmark.

| Landmark number | Type | Side | Description |
| --- | --- | --- | --- |
| 1 | fixed | Median | Medio-dorsal point of optic tectum |
| 2 | fixed | Median | Ventral-most point of hypothalamus dorsalis |
| 3 | fixed | Median | Dorsal posterior end of medulla oblongata |
| 4 | fixed | Median | Ventral point parallel to posterior end of medulla oblongata |
| 5 | fixed | Left | Left ventral border between forebrain bundle and hypothalamus dorsalis |
| 6 | fixed | Right | Right ventral border between forebrain bundle and hypothalamus dorsalis |
| 7 | fixed | Median | Medio-ventral point of optic chiasm |
| 8 | fixed | Left | Left lateral-most point of hypothalamus dorsalis |
| 9 | fixed | Right | Right lateral-most point of hypothalamus dorsalis |
| 10 | fixed | Median | Medio-dorsal point of anterior medulla oblongata |
| 11 | fixed | Left | Left dorsal-most point of telencephalon |
| 12 | fixed | Right | Right dorsal-most point of telencephalon |
| 13 | fixed | Left | Left anterior most point of olfactory bulb |
| 14 | curveSM | Left | Semilandmark in lateral region of left olfactory bulb |
| 15 | curveSM | Left | Semilandmark in lateral region of left olfactory bulb |
| 16 | fixed | Left | Left lateral and posterior end of olfactory bulb |
| 17 | curveSM | Left | Semilandmark in lateral region of left olfactory bulb |
| 18 | curveSM | Left | Semilandmark in lateral region of left olfactory bulb |
| 19 | fixed | Left | Left posterior end of telencephalon |
| 20 | fixed | Right | Right anterior most point of olfactory bulb |
| 21 | curveSM | Right | Semilandmark in lateral region of right olfactory bulb |
| 22 | curveSM | Right | Semilandmark in lateral region of right olfactory bulb |
| 23 | fixed | Right | Right lateral and posterior end of olfactory bulb |
| 24 | curveSM | Right | Semilandmark in lateral region of right olfactory bulb |
| 25 | curveSM | Right | Semilandmark in lateral region of right olfactory bulb |
| 26 | fixed | Right | Right posterior end of telencephalon |
| 27 | fixed | Left | Left lateral and anterior most point of white matter of medulla oblongata |
| 28 | curveSM | Left | Semilandmark on the left crest of white matter of medulla oblongata |
| 29 | curveSM | Left | Semilandmark on the left crest of white matter of medulla oblongata |
| 30 | fixed | Left | Left dorsal point at lateral constriction of medulla oblongata |
| 31 | fixed | Right | Right lateral and anterior most point of white matter of medulla oblongata |
| 32 | curveSM | Right | Semilandmark on the right crest of white matter of medulla oblongata |
| 33 | curveSM | Right | Semilandmark on the right crest of white matter of medulla oblongata |
| 34 | fixed | Right | Right dorsal point at lateral constriction of medulla oblongata |
| 35 | fixed | Left | Left border between tegmentum and hypothalamus dorsalis |
| 36 | curveSM | Left | Semilandmark in the lateral region of tegmentum |
| 37 | curveSM | Left | Semilandmark in the lateral region of tegmentum |
| 38 | curveSM | Left | Semilandmark in the lateral region of optic tectum |
| 39 | curveSM | Left | Semilandmark in the lateral region of optic tectum |
| 40 | curveSM | Left | Semilandmark in the posterior and dorsal region of cerebellum |
| 41 | fixed | Median | Medio-posterior point of cerebellum |
| 42 | curveSM | Right | Semilandmark in the posterior and dorsal region of cerebellum |
| 43 | curveSM | Right | Semilandmark in the lateral region of optic tectum |
| 44 | curveSM | Right | Semilandmark in the lateral region of optic tectum |
| 45 | curveSM | Right | Semilandmark in the lateral region of tegmentum |
| 46 | curveSM | Right | Semilandmark in the lateral region of tegmentum |
| 47 | fixed | Right | Right border between tegmentum and hypothalamus dorsalis |
| 48 | fixed | Left | Left lateral and anterior most point of grey matter of medulla oblongata |
| 49 | curveSM | Left | Semilandmark in the anterior region of grey matter of medulla oblongata |
| 50 | curveSM | Left | Semilandmark in the anterior region of grey matter of medulla oblongata |
| 51 | fixed | Median | Median ventral and anterior most point of grey matter of medulla oblongata |
| 52 | curveSM | Right | Semilandmark in the anterior region of grey matter of medulla oblongata |
| 53 | curveSM | Right | Semilandmark in the anterior region of grey matter of medulla oblongata |
| 54 | fixed | Right | Right lateral and anterior most point of grey matter of medulla oblongata |
| 55 | surfaceSM | Left | Semilandmark in the ventral region of forebrain bundle |
| 56 | surfaceSM | Left | Semilandmark in the ventral region of forebrain bundle |
| 57 | surfaceSM | Median | Semilandmark in the ventral region of forebrain bundle |
| 58 | surfaceSM | Right | Semilandmark in the ventral region of forebrain bundle |
| 59 | surfaceSM | Right | Semilandmark in the ventral region of forebrain bundle |
| 60 | surfaceSM | Left | Semilandmark in the ventral region of telencephalon |
| 61 | surfaceSM | Left | Semilandmark in the ventral region of telencephalon |
| 62 | surfaceSM | Left | Semilandmark in the ventral region of telencephalon |
| 63 | surfaceSM | Left | Semilandmark in the ventral region of olfactory bulb |
| 64 | surfaceSM | Left | Semilandmark in the ventral region of olfactory bulb |
| 65 | surfaceSM | Right | Semilandmark in the ventral region of telencephalon |
| 66 | surfaceSM | Right | Semilandmark in the ventral region of telencephalon |
| 67 | surfaceSM | Right | Semilandmark in the ventral region of telencephalon |
| 68 | surfaceSM | Right | Semilandmark in the ventral region of olfactory bulb |
| 69 | surfaceSM | Right | Semilandmark in the ventral region of olfactory bulb |
| 70 | surfaceSM | Left | Semilandmark in the dorsal region of dorsal pallium |
| 71 | surfaceSM | Left | Semilandmark in the dorsal region of dorsal pallium |
| 72 | surfaceSM | Left | Semilandmark in the dorsal region of olfactory bulb |
| 73 | surfaceSM | Left | Semilandmark in the dorsal region of olfactory bulb |
| 74 | surfaceSM | Left | Semilandmark in the lateral region of lateral pallium |
| 75 | surfaceSM | Left | Semilandmark in the lateral region of lateral pallium |
| 76 | surfaceSM | Left | Semilandmark in the lateral region of striatum |
| 77 | surfaceSM | Left | Semilandmark in the lateral region of striatum |
| 78 | surfaceSM | Right | Semilandmark in the lateral region of lateral pallium |
| 79 | surfaceSM | Right | Semilandmark in the lateral region of lateral pallium |
| 80 | surfaceSM | Right | Semilandmark in the lateral region of striatum |
| 81 | surfaceSM | Right | Semilandmark in the lateral region of striatum |
| 82 | surfaceSM | Right | Semilandmark in the ventral region of hypothalamus ventralis |
| 83 | surfaceSM | Right | Semilandmark in the ventral region of hypothalamus ventralis |
| 84 | surfaceSM | Left | Semilandmark in the ventral region of hypothalamus ventralis |
| 85 | surfaceSM | Left | Semilandmark in the ventral region of hypothalamus ventralis |
| 86 | surfaceSM | Right | Semilandmark in the lateral region of hypothalamus dorsalis |
| 87 | surfaceSM | Right | Semilandmark in the lateral region of hypothalamus dorsalis |
| 88 | surfaceSM | Right | Semilandmark in the lateral region of hypothalamus dorsalis |
| 89 | surfaceSM | Right | Semilandmark in the lateral region of hypothalamus dorsalis |
| 90 | surfaceSM | Right | Semilandmark in the lateral region of hypothalamus dorsalis |
| 91 | surfaceSM | Left | Semilandmark in the lateral region of hypothalamus dorsalis |
| 92 | surfaceSM | Left | Semilandmark in the lateral region of hypothalamus dorsalis |
| 93 | surfaceSM | Left | Semilandmark in the lateral region of hypothalamus dorsalis |
| 94 | surfaceSM | Left | Semilandmark in the lateral region of hypothalamus dorsalis |
| 95 | surfaceSM | Left | Semilandmark in the lateral region of hypothalamus dorsalis |
| 96 | surfaceSM | Left | Semilandmark in the lateral region of ventral thalamus |
| 97 | surfaceSM | Left | Semilandmark in the lateral region of ventral thalamus |
| 98 | surfaceSM | Left | Semilandmark in the lateral region of dorsal thalamus |
| 99 | surfaceSM | Left | Semilandmark in the lateral region of optic tectum |
| 100 | surfaceSM | Right | Semilandmark in the lateral region of ventral thalamus |
| 101 | surfaceSM | Right | Semilandmark in the lateral region of ventral thalamus |
| 102 | surfaceSM | Right | Semilandmark in the lateral region of dorsal thalamus |
| 103 | surfaceSM | Right | Semilandmark in the lateral region of optic tectum |
| 104 | surfaceSM | Left | Semilandmark in the lateral region of tegmentum |
| 105 | surfaceSM | Left | Semilandmark in the lateral region of tegmentum |
| 106 | surfaceSM | Left | Semilandmark in the lateral region of tegmentum |
| 107 | surfaceSM | Left | Semilandmark in the dorsal region of optic tectum |
| 108 | surfaceSM | Left | Semilandmark in the dorsal region of optic tectum |
| 109 | surfaceSM | Right | Semilandmark in the lateral region of tegmentum |
| 110 | surfaceSM | Right | Semilandmark in the lateral region of tegmentum |
| 111 | surfaceSM | Right | Semilandmark in the lateral region of tegmentum |
| 112 | surfaceSM | Right | Semilandmark in the dorsal region of optic tectum |
| 113 | surfaceSM | Right | Semilandmark in the dorsal region of optic tectum |
| 114 | surfaceSM | Left | Semilandmark in the dorsal region of optic tectum |
| 115 | surfaceSM | Left | Semilandmark in the dorsal region of optic tectum |
| 116 | surfaceSM | Right | Semilandmark in the dorsal region of optic tectum |
| 117 | surfaceSM | Right | Semilandmark in the dorsal region of optic tectum |
| 118 | surfaceSM | Left | Semilandmark in the anterior region of white matter of medulla oblongata |
| 119 | surfaceSM | Left | Semilandmark in the anterior region of white matter of medulla oblongata |
| 120 | surfaceSM | Right | Semilandmark in the anterior region of white matter of medulla oblongata |
| 121 | surfaceSM | Right | Semilandmark in the anterior region of white matter of medulla oblongata |
| 122 | surfaceSM | Left | Semilandmark in the anterior region of white matter of medulla oblongata |
| 123 | surfaceSM | Left | Semilandmark in the anterior region of white matter of medulla oblongata |
| 124 | surfaceSM | Right | Semilandmark in the anterior region of white matter of medulla oblongata |
| 125 | surfaceSM | Right | Semilandmark in the anterior region of white matter of medulla oblongata |
| 126 | surfaceSM | Left | Semilandmark in the dorsal region of white matter of medulla oblongata at lateral constriction |
| 127 | surfaceSM | Right | Semilandmark in the dorsal region of white matter of medulla oblongata at lateral constriction |
| 128 | surfaceSM | Left | Semilandmark in the posterior region of white matter of medulla oblongata |
| 129 | surfaceSM | Left | Semilandmark in the posterior region of white matter of medulla oblongata |
| 130 | surfaceSM | Left | Semilandmark in the posterior region of white matter of medulla oblongata |
| 131 | surfaceSM | Left | Semilandmark in the posterior region of white matter of medulla oblongata |
| 132 | surfaceSM | Right | Semilandmark in the posterior region of white matter of medulla oblongata |
| 133 | surfaceSM | Right | Semilandmark in the posterior region of white matter of medulla oblongata |
| 134 | surfaceSM | Right | Semilandmark in the posterior region of white matter of medulla oblongata |
| 135 | surfaceSM | Right | Semilandmark in the posterior region of white matter of medulla oblongata |
| 136 | surfaceSM | Left | Semilandmark in the lateral and anterior region of grey matter of medulla oblongata |
| 137 | surfaceSM | Left | Semilandmark in the lateral and anterior region of grey matter of medulla oblongata |
| 138 | surfaceSM | Left | Semilandmark in the lateral and posterior region of grey matter of medulla oblongata |
| 139 | surfaceSM | Left | Semilandmark in the lateral and posterior region of grey matter of medulla oblongata |
| 140 | surfaceSM | Left | Semilandmark in the lateral and posterior region of grey matter of medulla oblongata |
| 141 | surfaceSM | Right | Semilandmark in the lateral and anterior region of grey matter of medulla oblongata |
| 142 | surfaceSM | Right | Semilandmark in the lateral and anterior region of grey matter of medulla oblongata |
| 143 | surfaceSM | Right | Semilandmark in the lateral and posterior region of grey matter of medulla oblongata |
| 144 | surfaceSM | Right | Semilandmark in the lateral and posterior region of grey matter of medulla oblongata |
| 145 | surfaceSM | Right | Semilandmark in the lateral and posterior region of grey matter of medulla oblongata |
| 146 | surfaceSM | Median | Semilandmark in the median and anterior region of grey matter of medulla oblongata |
| 147 | surfaceSM | Median | Semilandmark in the median and anterior region of grey matter of medulla oblongata |
| 148 | surfaceSM | Median | Semilandmark in the median and posterior region of grey matter of medulla oblongata |
| 149 | surfaceSM | Median | Semilandmark in the median and posterior region of grey matter of medulla oblongata |
| 150 | surfaceSM | Median | Semilandmark in the median and posterior region of grey matter of medulla oblongata |
| 151 | surfaceSM | Right | Semilandmark in the dorsal region of dorsal pallium |
| 152 | surfaceSM | Right | Semilandmark in the dorsal region of dorsal pallium |
| 153 | surfaceSM | Right | Semilandmark in the dorsal region of olfactory bulb |
| 154 | surfaceSM | Right | Semilandmark in the dorsal region of olfactory bulb |
| 155 | surfaceSM | Left | Semilandmark in the ventral region of optic chiasm |
| 156 | surfaceSM | Left | Semilandmark in the ventral region of optic chiasm |
| 157 | surfaceSM | Left | Semilandmark in the lateral region of optic chiasm |
| 158 | surfaceSM | Left | Semilandmark in the lateral and anterior region of hypothalamus dorsalis |
| 159 | surfaceSM | Left | Semilandmark in the lateral and anterior region of hypothalamus dorsalis |
| 160 | surfaceSM | Median | Semilandmark in the median and posterior region of optic chiasm |
| 161 | surfaceSM | Median | Semilandmark in the medio-ventral region of hypothalamus dorsalis |
| 162 | surfaceSM | Right | Semilandmark in the ventral region of optic chiasm |
| 163 | surfaceSM | Right | Semilandmark in the ventral region of optic chiasm |
| 164 | surfaceSM | Right | Semilandmark in the lateral region of optic chiasm |
| 165 | surfaceSM | Right | Semilandmark in the lateral and anterior region of hypothalamus dorsalis |
| 166 | surfaceSM | Right | Semilandmark in the lateral and anterior region of hypothalamus dorsalis |
| 167 | surfaceSM | Left | Semilandmark in the dorso-lateral border of hypothalamus dorsalis and ventral thalamus |
| 168 | surfaceSM | Left | Semilandmark in the dorso-lateral border of hypothalamus dorsalis and ventral thalamus |
| 169 | surfaceSM | Right | Semilandmark in the dorso-lateral border of hypothalamus dorsalis and ventral thalamus |
| 170 | surfaceSM | Right | Semilandmark in the dorso-lateral border of hypothalamus dorsalis and ventral thalamus |
| 171 | surfaceSM | Left | Semilandmark in the lateral region of ventral thalamus |
| 172 | surfaceSM | Left | Semilandmark in the lateral region of ventral thalamus |
| 173 | surfaceSM | Left | Semilandmark in the lateral region of dorsal thalamus |
| 174 | surfaceSM | Median | Semilandmark in the dorsal region of dorsal thalamus |
| 175 | surfaceSM | Right | Semilandmark in the lateral region of dorsal thalamus |
| 176 | surfaceSM | Right | Semilandmark in the lateral region of ventral thalamus |
| 177 | surfaceSM | Right | Semilandmark in the lateral region of ventral thalamus |
| 178 | surfaceSM | Median | Semilandmark in the dorsal region of dorsal thalamus |
| 179 | surfaceSM | Median | Semilandmark in the dorsal region of dorsal thalamus |
| 180 | surfaceSM | Median | Semilandmark in the dorsal region of optic tectum |
| 181 | surfaceSM | Median | Semilandmark in the dorsal region of optic tectum |


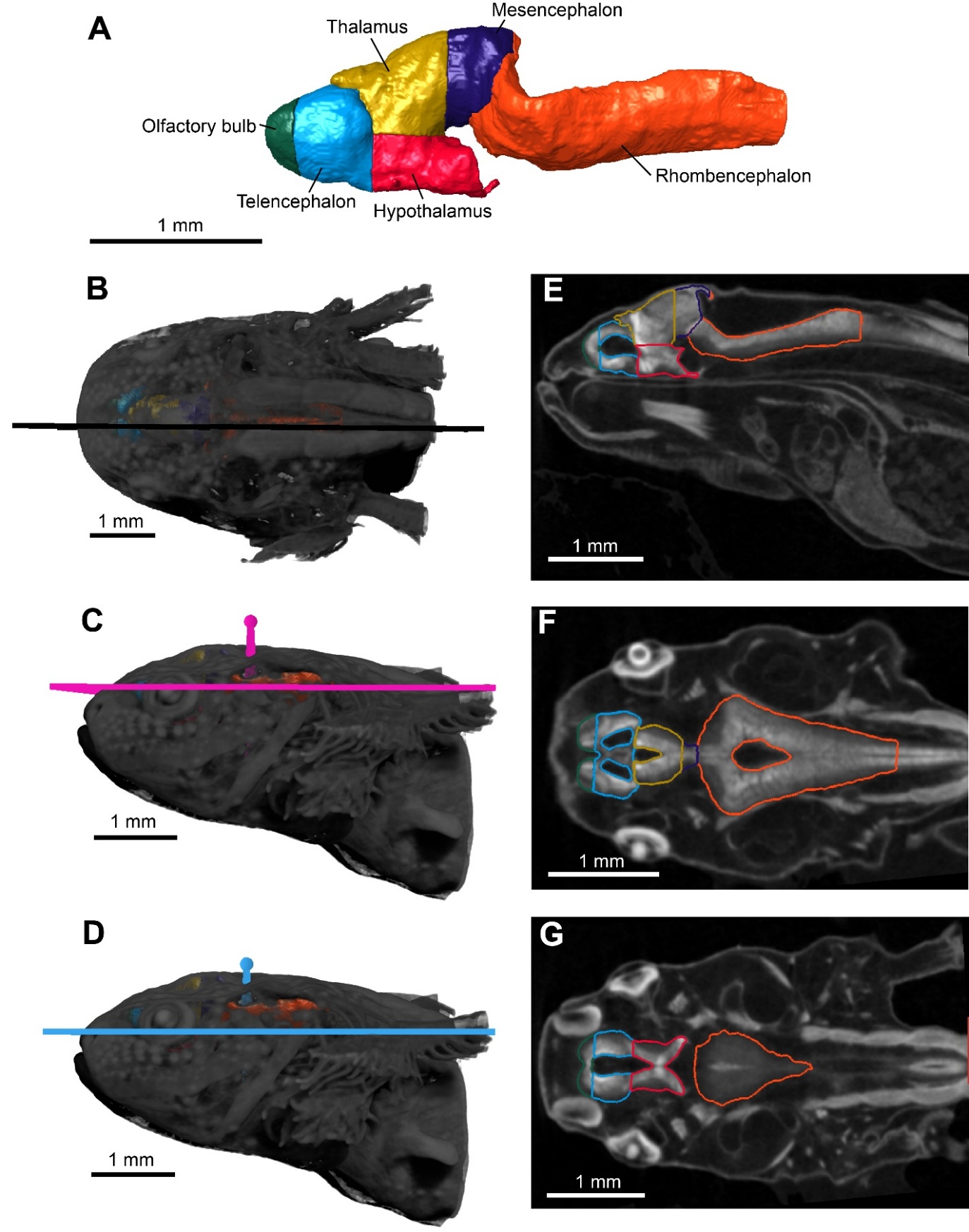


Figure 1. Representation of delineation of volume partitions from a 3D point of view (A) and from CT images of different parts of a specimen (B, C, D, E, F, G). A sagittal view near the median section of the specimen is shown (E) and a black line represent the area associated with that view (B). Also, two transversal views are presented (F and G) and the area corresponding to these views are indicated by a pink (C) and blue lines (D), respectively.

Table 2. Size-included Procrustes ANOVA and pairwise distance between least-squares means and shape variance comparisons results for the size-included brain shape of *A. mexicanum* larvae. The first line presents the Procrustes ANOVA results and the subsequent lines present the effect size (Z) of the pairwise distance between means and shape variance comparisons. P-values from the pairwise comparisons are in parentheses. Bold values represent significant results.

|  | R^2^ | F | Z | P-value |
| --- | --- | --- | --- | --- |
| ANOVA | 0.612 | 38.439 | 5.469 | **0.001** |
| Procrustes distances | | | | |
| Developmental stage | 47 | 50 | 52 | 54 |
| 50 | 3.088 (**0.001**) |  |  |  |
| 52 | 3.434 (**0.001**) | 1.003 (0.195) |  |  |
| 54 | 3.283 (**0.001**) | 1.647 (0.051) | 0.598 (0.298) |  |
| Variance | | | | |
| Developmental stage | 47 | 50 | 52 | 54 |
| 50 | 0.910 (0.199) |  |  |  |
| 52 | -1.585 (0.933) | 0.762 (0.251) |  |  |
| 54 | -0.878 (0.799) | 1,169 (0.135) | -0.687 (0.742) |  |

Table 3. Size-excluded Procrustes ANOVA and pairwise distance between least-squares means and shape variance comparisons results for the size-excluded brain shape of *A. mexicanum* larvae. The first line present the Procrustes ANOVA results and the subsequent lines present the effect size (Z) of the pairwise distance between means and shape variance comparisons. P-values from the pairwise comparisons are in parentheses. Bold values represent significant results.

|  | R^2^ | F | Z | P-value |
| --- | --- | --- | --- | --- |
| ANOVA | 0.252 | 8.214 | 4.665 | **0.001** |
| Procrustes distances | | | | |
| Developmental stage | 47 | 50 | 52 | 54 |
| 50 | 4.326 (**0.001**) |  |  |  |
| 52 | 3.216 (**0.001**) | 2.124 (**0.015**) |  |  |
| 54 | 1.542 (0.065) | 3.189 (**0.001**) | 2.082 (**0.021**) |  |
| Variance | | | | |
| Developmental stage | 47 | 50 | 52 | 54 |
| 50 | 0.345 (0.379) |  |  |  |
| 52 | -1.023 (0.834) | 0.639 (0.284) |  |  |
| 54 | 0.396 (0.368) | 1.407 (0.088) | 0.026 (0.489) |  |


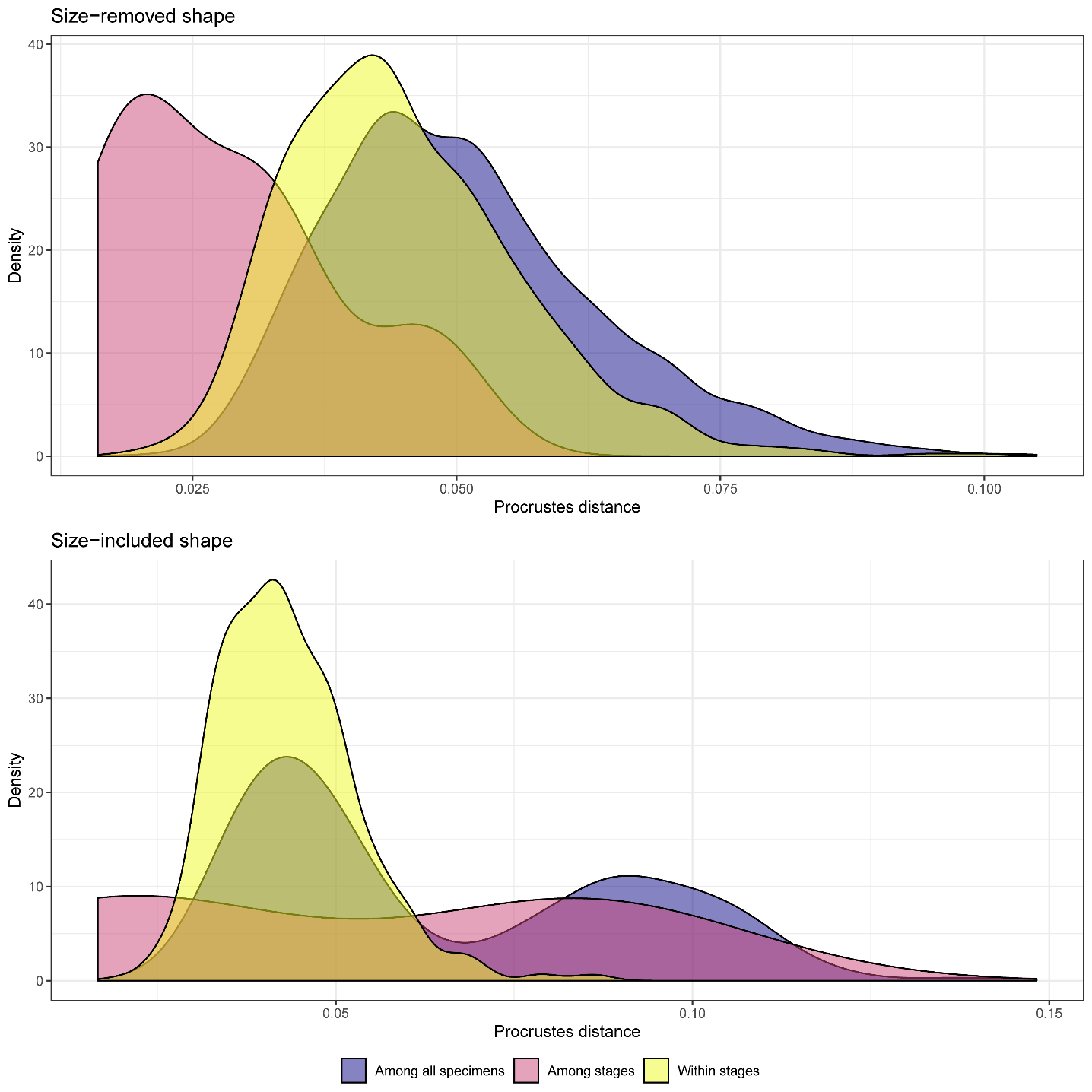


Figure 2. Density plots of the frequency of Procrustes distances associated with among stages variation, within stages variation and variation among all specimens of *A. mexicanum* larvae.


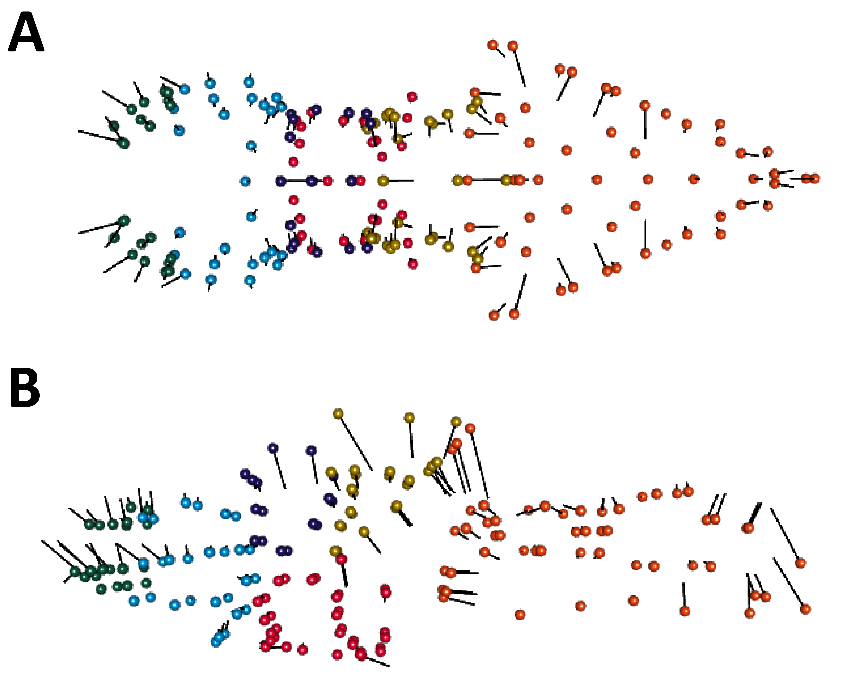


Figure 3. Dorsal (A) and lateral (B) views of the shape variation from the Procrustes linear regression. Colors represent the six different brain regions of *A. mexicanum* larvae: olfactory bulb (dark green), telencephalon (light blue), thalamus (dark purple), hypothalamus (red), mesencephalon (gold), and rhombencephalon (orange).


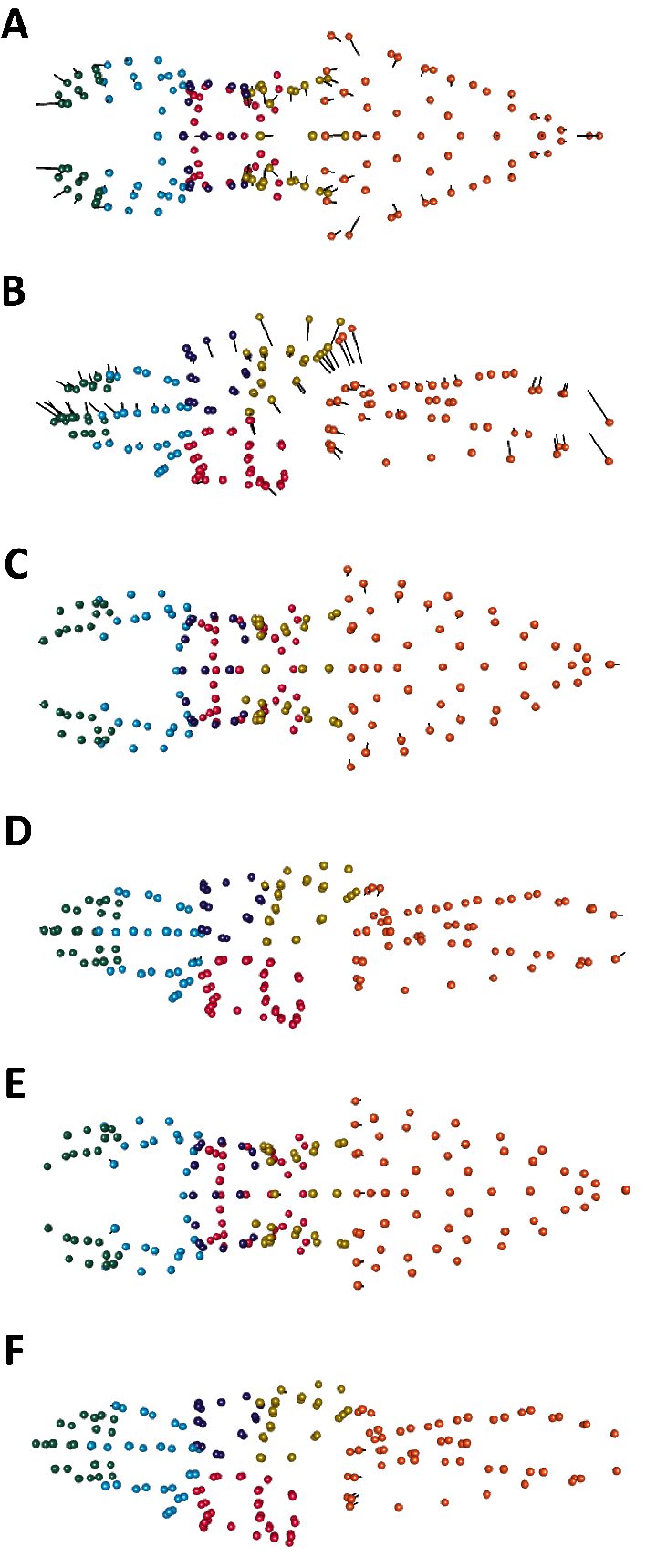


Figure 4. Dorsal and lateral views of the shape variation from the size-included dataset between the mean shape of stage 47 and stage 50 (A, B), the mean shape of stage 50 and 52 (C, D), and the mean shape of stage 52 and 54 (E, F) of *A. mexicanum* larvae. Colors represent the six different brain regions: olfactory bulb (dark green), telencephalon (light blue), thalamus (dark purple), hypothalamus (red), mesencephalon (gold), and rhombencephalon (orange).


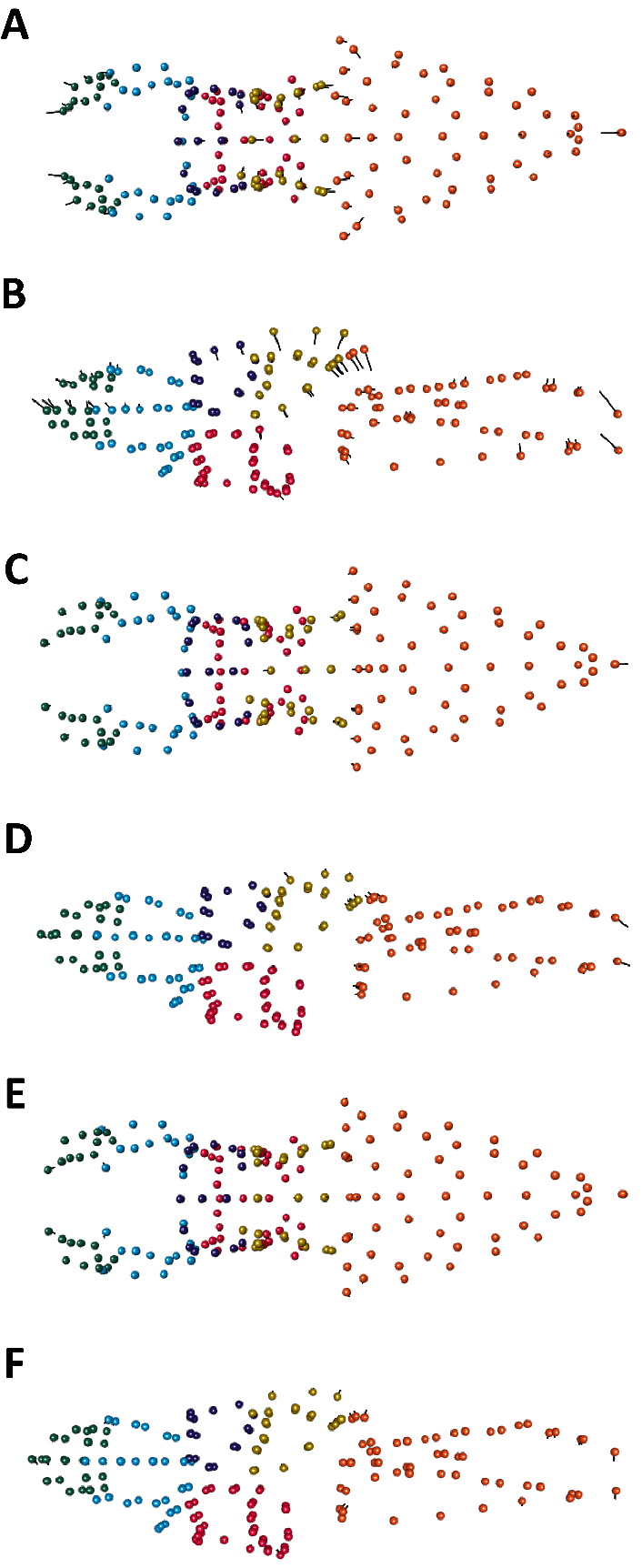


Figure 5. Dorsal and lateral views of the shape variation from the size-removed dataset between the mean shape of stage 47 and stage 50 (A, B), the mean shape of stage 50 and 52 (C, D), and the mean shape of stage 52 and 54 (E, F) of *A. mexicanum* larvae. Colors represent the six different brain regions: olfactory bulb (dark green), telencephalon (light blue), thalamus (dark purple), hypothalamus (red), mesencephalon (gold), and rhombencephalon (orange).

Table 4. Extra sum of squares F-test results and AIC values for the allometric model selection of brain volumetric data for *A. mexicanum* larvae. Bold values indicate that the piecewise model had a significantly better fit to the data.

| Brain region | extra sum of squares F-test | | Linear model AIC | Piecewise model AIC |
| --- | --- | --- | --- | --- |
|  | F | P-value |  |  |
| Olfactory bulbs | 6.311 | **0.003** | -166.925 | -175.206 |
| Telencephalon | 0.647 | 0.527 | -259.610 | -256.962 |
| Hypothalamus | 0.898 | 0.412 | -242.553 | -240.424 |
| Thalamus | 6.693 | **0.002** | -272.980 | -281.944 |
| Mesencephalon | 4.815 | **0.011** | -225.429 | -230.969 |
| Rhombencephalon | 1.926 | 0.153 | -326.192 | -326.151 |

Table 5. Coefficients (± 95% confidence interval) associated with the best supported regression model of brain region volumes of *A. mexicanum* larvae. Each line present results of a different model.

| Brain region | Best supported regression model | Slope | Intercept | R^2^ |
| --- | --- | --- | --- | --- |
| Olfactory bulbs | Piecewise | slope 1: 2.184 ± 0.678  slope 2: 0.964± 0.309 | -1.128 ± 0.122 | 0.777 |
| Telencephalon | Linear | 1.611 ± 0.107 | -0.799 ± 0.010 | 0.922 |
| Thalamus | Piecewise | slope 1: 0.641± 0.195  slope 2: 1.186 ± 0.225 | -0.925 ± 0.037 | 0.867 |
| Hypothalamus | Linear | 0.888 ± 0.106 | -1.114 ± 0.011 | 0.785 |
| Mesencephalon | Piecewise | slope 1: 1.570 ± 0.420  slope 2: 0.883 ± 0.229 | -0.833 ± 0.084 | 0.837 |
| Rhombencephalon | Linear | 0.638 ± 0.052 | -0.398 ± 0.006 | 0.886 |

Table 6. Results of morphological integration analyses using partial least squares (PLS) and modularity analyses using the covariance ratio (CR) and EMMLi methods for the six *a priori* hypotheses for each developmental stage of brain shape of *A. mexicanum* larvae. The results in bold represent best supported hypotheses.

|  | CR method | | | EMMLi | | | Morphological integration | | | Stage |
| --- | --- | --- | --- | --- | --- | --- | --- | --- | --- | --- |
| Hypothesis | CR | P-value | Z | K | AICc | ΔAICc | PLS | P-value | Z |  |
| 1 | 0.941 | 0.024 | -1.878 | 4 | 181242.440 | 40463.639 | 0.957 | 0.001 | 3.802 | 47 |
| 2 | 0.882 | 0.001 | -2.840 | 4 | 170171.452 | 29392.650 | 0.877 | 0.011 | 2.345 |  |
| 3 | 0.839 | 0.001 | -4.397 | 7 | 165138.557 | 24359.756 | 0.869 | 0.001 | 3.209 |  |
| 4 | 0.802 | 0.001 | -5.780 | 11 | 147769.785 | 6990.983 | 0.873 | 0.001 | 4.535 |  |
| 5 | 0.758 | 0.001 | -6.731 | 16 | 145813.314 | 5034.512 | 0.846 | 0.001 | 5.212 |  |
| **6** | **0.712** | **0.001** | **-7.466** | **22** | **140778.801** | **0.000** | **0.798** | **0.001** | **4.839** |  |
| 1 | 0.889 | 0.001 | -3.537 | 4 | 173655.567 | 41521.308 | 0.907 | 0.001 | 2.730 | 50 |
| 2 | 0.806 | 0.001 | -4.025 | 4 | 162970.205 | 30835.946 | 0.844 | 0.020 | 2.085 |  |
| 3 | 0.774 | 0.001 | -5.653 | 7 | 156852.864 | 24718.605 | 0.814 | 0.001 | 3.126 |  |
| 4 | 0.754 | 0.001 | -6.730 | 11 | 139166.433 | 7032.174 | 0.813 | 0.001 | 3.723 |  |
| 5 | 0.731 | 0.001 | -6.948 | 16 | 137349.728 | 5215.469 | 0.806 | 0.001 | 4.248 |  |
| **6** | **0.728** | **0.001** | **-6.914** | **22** | **132134.259** | **0.000** | **0.810** | **0.001** | **4.486** |  |
| 1 | 0.885 | 0.001 | -5.087 | 4 | 156246.603 | 37155.987 | 0.904 | 0.002 | 2.661 | 52 |
| 2 | 0.807 | 0.001 | -5.350 | 4 | 146676.074 | 27585.458 | 0.853 | 0.025 | 2.005 |  |
| 3 | 0.799 | 0.001 | -6.881 | 7 | 141566.624 | 22476.008 | 0.852 | 0.001 | 3.626 |  |
| 4 | 0.753 | 0.001 | -8.665 | 11 | 125361.243 | 6270.627 | 0.811 | 0.001 | 4.121 |  |
| 5 | 0.762 | 0.001 | -8.315 | 16 | 123685.084 | 4594.468 | 0.811 | 0.001 | 4.473 |  |
| **6** | **0.733** | **0.001** | **-8.389** | **22** | **119090.616** | **0.000** | **0.792** | **0.001** | **4.398** |  |
| 1 | 0.937 | 0.003 | -2.555 | 4 | 179548.179 | 41095.403 | 0.944 | 0.001 | 3.516 | 54 |
| 2 | 0.845 | 0.001 | -4.345 | 4 | 167831.260 | 29378.485 | 0.939 | 0.001 | 3.639 |  |
| 3 | 0.840 | 0.001 | -5.421 | 7 | 162487.849 | 24035.073 | 0.930 | 0.001 | 4.169 |  |
| 4 | 0.781 | 0.001 | -7.507 | 11 | 145370.834 | 6918.058 | 0.879 | 0.001 | 3.862 |  |
| 5 | 0.769 | 0.001 | -7.802 | 16 | 143484.421 | 5031.645 | 0.883 | 0.001 | 4.067 |  |
| **6** | **0.757** | **0.001** | **-7.934** | **22** | **138452.776** | **0.000** | **0.855** | **0.001** | **4.268** |  |

Table 7. P-values from pairwise covariance ratio effect sizes associated with modularity hypotheses for brain shape of *A. mexicanum* larvae. Bold values represent significant results.

| Modularity hypothesis | No modules | 1 | 2 | 3 | 4 | 5 | Stage |
| --- | --- | --- | --- | --- | --- | --- | --- |
| 1 | **0.000** |  |  |  |  |  | All |
| 2 | **0.000** | 0.462 |  |  |  |  |  |
| 3 | **0.000** | 0.336 | 0.323 |  |  |  |  |
| 4 | **0.000** | **0.039** | 0.056 | 0.098 |  |  |  |
| 5 | **0.000** | **0.012** | **0.022** | **0.037** | 0.287 |  |  |
| 6 | **0.000** | **0.005** | **0.010** | **0.016** | 0.162 | 0.329 |  |
| 1 | **0.030** |  |  |  |  |  | 47 |
| 2 | **0.002** | 0.192 |  |  |  |  |  |
| 3 | **0.000** | 0.062 | 0.298 |  |  |  |  |
| 4 | **0.000** | **0.019** | 0.165 | 0.316 |  |  |  |
| 5 | **0.000** | **0.006** | 0.084 | 0.175 | 0.314 |  |  |
| 6 | **0.000** | **0.002** | **0.043** | 0.093 | 0.184 | 0.337 |  |
| 1 | **0.000** |  |  |  |  |  | 50 |
| 2 | **0.000** | 0.160 |  |  |  |  |  |
| 3 | **0.000** | 0.072 | 0.394 |  |  |  |  |
| 4 | **0.000** | **0.041** | 0.326 | 0.422 |  |  |  |
| 5 | **0.000** | **0.027** | 0.271 | 0.350 | 0.419 |  |  |
| 6 | **0.000** | **0.027** | 0.267 | 0.345 | 0.413 | 0.494 |  |
| 1 | **0.000** |  |  |  |  |  | 52 |
| 2 | **0.000** | 0.086 |  |  |  |  |  |
| 3 | **0.000** | **0.049** | 0.471 |  |  |  |  |
| 4 | **0.000** | **0.008** | 0.271 | 0.269 |  |  |  |
| 5 | **0.000** | **0.011** | 0.294 | 0.296 | 0.469 |  |  |
| 6 | **0.000** | **0.004** | 0.197 | 0.187 | 0.377 | 0.350 |  |
| 1 | **0.005** |  |  |  |  |  | 54 |
| 2 | **0.000** | **0.045** |  |  |  |  |  |
| 3 | **0.000** | **0.031** | 0.478 |  |  |  |  |
| 4 | **0.000** | **0.003** | 0.255 | 0.203 |  |  |  |
| 5 | **0.000** | **0.001** | 0.181 | 0.131 | 0.375 |  |  |
| 6 | **0.000** | **0.001** | 0.132 | 0.087 | 0.280 | 0.394 |  |


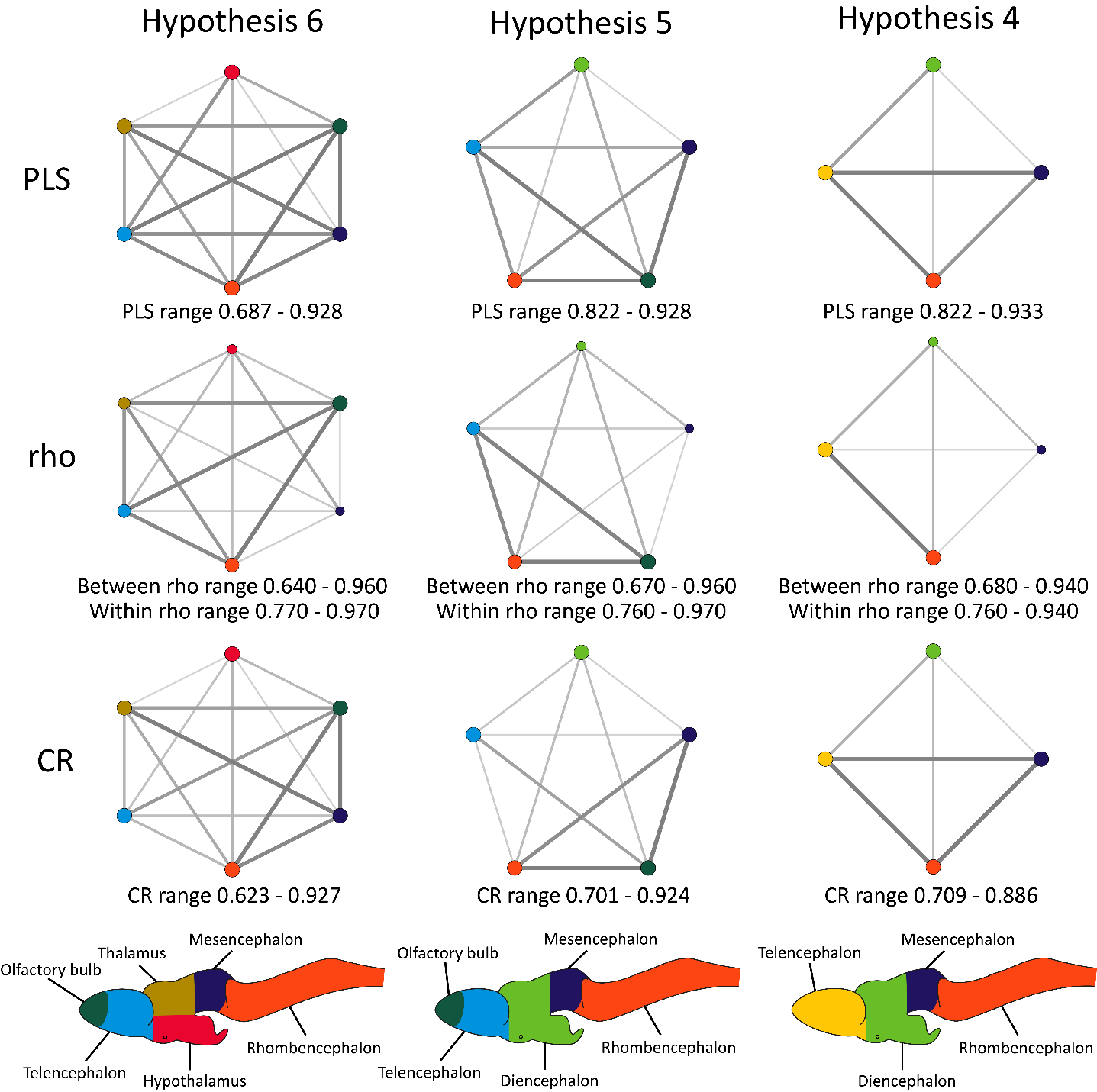


Figure 6. Network representations of the strength of morphological integration from the covariance ratio (CR), the EMMLi (rho) and the partial least squares (PLS) analyses for three *a priori* hypotheses for the brain shape of *A. mexicanum* larvae. Edge width in each network represents the strength of the association between two modules. Note that the edge width is based on values within each analysis (i.e., not directly comparable between networks). For the EMMLi analysis, the edge width represents the correlation between two modules and the size of nodes represents the within module correlation.

Table 8. Global integration results for each studied developmental stage of *A. mexicanum* larvae.

| Stage | Global integration slope |
| --- | --- |
| All | -0.865 |
| 47 | -0.743 |
| 50 | -0.821 |
| 52 | -0.892 |
| 54 | -0.881 |

Table 9. P-values from the pairwise comparison of the effect size of relative eigenvalue index comparing differences in integration level among studied developmental stages of *A. mexicanum* larvae.

| Stage | 47 | 50 | 52 |
| --- | --- | --- | --- |
| 50 | 0.841 |  |  |
| 52 | 0.907 | 0.757 |  |
| 54 | 0.452 | 0.347 | 0.541 |

Table 10. P-values from the pairwise comparison of the effect size of the pairwise PLS values of each modularity hypothesis for the brain shape of *A. mexicanum* larvae. Bold values represent significant results.

| Hypothesis | 1 | 2 | 3 | 4 | 5 |
| --- | --- | --- | --- | --- | --- |
| 2 | **0.033** |  |  |  |  |
| 3 | **0.034** | **0.042** |  |  |  |
| 4 | **0.048** | **0.047** | 0.388 |  |  |
| 5 | **0.017** | **0.012** | 0.096 | 0.181 |  |
| 6 | **0.035** | **0.020** | 0.181 | 0.285 | 0.365 |
