# Supplementary material for: Exploring Larval Axolotl Brain Development: Insights Into Developmental and Functional Constraints
Source: Evol Dev. 2026 Mar 8;28(1):e70034. doi: 10.1111/ede.70034 (PMC12968592; doi:10.1111/ede.70034)
Supplement: Supplementary file 3 — Supplemental results 3. [file EDE-28-e70034-s001.docx]

**Title**

Exploring Larval Axolotl Brain Development: Insights into Developmental and Functional Constraints

**Authors**

Laurent Houle^1*^, Olivier Larouche^2^ and Richard Cloutier^1,3^

**Affiliations**

^1^ Laboratoire de Paléontologie et Biologie évolutive, Université du Québec à Rimouski, 300 Allée des Ursulines, Rimouski, Quebec G5L 3A1

^2^ Biology Department, Western Carolina University, Cullowhee, NC, 28723

^3^ Center of Excellence on the Evolution of Life, Basin Studies and Applied Paleontology; Paleontological Research and Education Center, Mahasarakham University, Maha Sarakham 44150, Thailand

^*^Corresponding author: [laurent_houle@uqar.ca](mailto:laurent_houle@uqar.ca)

Supporting information

**Supporting text for modular hypotheses justifications**

The first hypothesis follows ideas discussed in Ollonen et al. (2024), which highlight distinct developmental and topological behaviours of dorsal and ventral brain regions in squamates, with dorsal regions exhibiting greater variability and ventral regions maintaining more conservative trajectories. This pattern echoes the well-established dorsoventral organization of the vertebrate brain, in which dorsal and ventral domains correspond to genetically and developmentally distinct territories (Butler & Hodos, 2005; Jessell, 2000; Puelles, 2013; Puelles & Rubenstein, 2003).

The second hypothesis builds on evidence for the partial developmental and functional independence of the midbrain relative to both the forebrain and hindbrain. The mesencephalon forms a cohesive visuomotor integration center composed of the tectum and tegmentum, supporting orienting reflexes, multimodal integration, and motor gating (Butler & Hodos, 2005; Roth et al., 1997; Striedter, 2005). Its identity is established and maintained by the isthmic organizer (IsO), which imposes a sharp developmental boundary through FGF8–Wnt signalling (Kiecker & Lumsden, 2005; Rhinn & Brand, 2001).

The third hypothesis is based on the classical tripartite organization of the vertebrate brain, where the forebrain, midbrain, and hindbrain arise from distinct embryonic compartments separated by stable molecular and morphogenetic boundaries (Puelles & Rubenstein, 2003; Redies & Puelles, 2001). These territories are patterned by independent signaling centers that regulate regional identity and growth (Kiecker & Lumsden, 2005).

The fourth hypothesis aligns with the neuromeric model, which subdivides the forebrain into telencephalic and diencephalic domains, in addition to the mesencephalon and rhombencephalon. These four regions constitute discrete embryonic divisions that are molecularly delimited, developmentally autonomous, and preserved as coherent radial units into adulthood (Nieuwenhuys et al., 2014; Redies & Puelles, 2001).

The fifth hypothesis further separates the olfactory bulb from the remainder of the telencephalon based on its distinct functional and developmental properties. The olfactory bulb acts as a primary sensory system devoted to odor detection and early signal transformation and relies on specialized circuitry and direct sensory input that is largely segregated from higher-order telencephalic processing (Butler & Hodos, 2005; Corfield et al., 2015). In contrast, the rest of the telencephalon integrates multisensory information, supports associative and spatial processing, and scales more predictably with overall brain size following conserved developmental patterns in some vertebrates (Finlay & Darlington, 1995; Finlay et al., 2001). Comparative studies show that olfactory bulb size can vary independently from total telencephalic volume and can reflect ecological reliance on olfaction rather than overall brain enlargement (Gonzalez-Voyer et al., 2009; Yopak et al., 2010).

The sixth hypothesis retains the major developmental divisions of the brain while separately modelling regions with strongly divergent functional roles. The olfactory bulb functions as a dedicated sensory processor with evolutionary and developmental scaling patterns often decoupled from those of the remaining telencephalon (Corfield et al., 2015; Finlay & Darlington, 1995; Finlay et al., 2001; Gonzalez-Voyer et al., 2009; Yopak et al., 2010). The telencephalon supports higher-order associative and spatial processing (Striedter, 2005), while within the diencephalon, the thalamus acts as the principal sensory relay system and the hypothalamus regulates neuroendocrine function, homeostasis, and motivational behaviours (Butler & Hodos, 2005; Northcutt, 2002). The midbrain forms a cohesive visuomotor integration center, and the rhombencephalon coordinates motor pattern generation, balance, and autonomic control (Butler & Hodos, 2005; Nieuwenhuys et al., 2014; Northcutt, 2002). Together, these marked functional dissociations support modelling the olfactory bulb, telencephalon, thalamus, hypothalamus, mesencephalon, and rhombencephalon as six distinct modules.

**References**

Butler, A. B., & Hodos, W. (2005). *Comparative vertebrate neuroanatomy: evolution and adaptation*. John Wiley & Sons.

Corfield, J. R., Price, K., Iwaniuk, A. N., Gutiérrez-Ibáñez, C., Birkhead, T., & Wylie, D. R. (2015). Diversity in olfactory bulb size in birds reflects allometry, ecology, and phylogeny. *Frontiers in Neuroanatomy*, *9*, 102. <https://doi.org/https://doi.org/10.3389/fnana.2015.00102>

Finlay, B. L., & Darlington, R. B. (1995). Linked regularities in the development and evolution of mammalian brains. *Science*, *268*(5217), 1578-1584. <https://doi.org/10.1126/science.7777856>

Finlay, B. L., Darlington, R. B., & Nicastro, N. (2001). Developmental structure in brain evolution. *Behavioral and Brain Sciences*, *24*(2), 263-278. <https://doi.org/https://doi.org/10.1017/S0140525X01003958>

Gonzalez-Voyer, A., Winberg, S., & Kolm, N. (2009). Brain structure evolution in a basal vertebrate clade: evidence from phylogenetic comparative analysis of cichlid fishes. *BMC evolutionary biology*, *9*, 1-12. <https://doi.org/https://doi.org/10.1186/1471-2148-9-238>

Jessell, T. M. (2000). Neuronal specification in the spinal cord: inductive signals and transcriptional codes. *Nature Reviews Genetics*, *1*(1), 20-29.

Kiecker, C., & Lumsden, A. (2005). Compartments and their boundaries in vertebrate brain development. *Nature Reviews Neuroscience*, *6*(7), 553-564.

Nieuwenhuys, R., Ten Donkelaar, H. J., & Nicholson, C. (2014). *The central nervous system of vertebrates*. Springer.

Northcutt, R. G. (2002). Understanding vertebrate brain evolution. *Integrative and Comparative Biology*, *42*(4), 743-756.

Ollonen, J., Khannoon, E. R., Macrì, S., Vergilov, V., Kuurne, J., Saarikivi, J., Soukainen, A., Aalto, I.-M., Werneburg, I., & Diaz Jr, R. E. (2024). Dynamic evolutionary interplay between ontogenetic skull patterning and whole-head integration. *Nature ecology & evolution*, *8*(3), 536-551. <https://doi.org/https://doi.org/10.1038/s41559-023-02295-3>

Puelles, L. (2013). Plan of the developing vertebrate nervous system. *Pattering and Cell Type Specification in the Developing CNS and PNS*, *1*, 187-209.

Puelles, L., & Rubenstein, J. L. (2003). Forebrain gene expression domains and the evolving prosomeric model. *Trends in neurosciences*, *26*(9), 469-476.

Redies, C., & Puelles, L. (2001). Modularity in vertebrate brain development and evolution. *Bioessays*, *23*(12), 1100-1111. <https://doi.org/https://doi.org/10.1002/bies.10014>

Rhinn, M., & Brand, M. (2001). The midbrain–hindbrain boundary organizer. *Current opinion in neurobiology*, *11*(1), 34-42.

Roth, G., Nishikawa, K. C., & Wake, D. B. (1997). Genome size, secondary simplification, and the evolution of the brain in salamanders. *Brain, Behavior and Evolution*, *50*(1), 50-59. <https://doi.org/https://doi.org/10.1159/000113321>

Striedter, G. F. (2005). *Principles of Brain Evolution*. Sinauer associates.

Yopak, K. E., Lisney, T. J., Darlington, R. B., Collin, S. P., Montgomery, J. C., & Finlay, B. L. (2010). A conserved pattern of brain scaling from sharks to primates. *Proceedings of the National Academy of Sciences*, *107*(29), 12946-12951. <https://doi.org/https://doi.org/10.1073/pnas.1002195107>
